# Global Landscape and Dynamics of Parkin and USP30-Dependent Ubiquitylomes in iNeurons during Mitophagic Signaling

**DOI:** 10.1016/j.molcel.2019.11.013

**Published:** 2020-03-05

**Authors:** Alban Ordureau, Joao A. Paulo, Jiuchun Zhang, Heeseon An, Kirby N. Swatek, Joe R. Cannon, Qiaoqiao Wan, David Komander, J. Wade Harper

**Affiliations:** 1 Department of Cell Biology, Harvard Medical School, 240 Longwood Avenue, Boston, MA 02115, USA; 2 Department of Molecular Machines and Signaling, Max Planck Institute of Biochemistry, Martinsried 82152, Germany; 3 Medical Research Council Laboratory of Molecular Biology, Francis Crick Avenue, Cambridge CB2 0QH, UK; 4 Ubiquitin Signalling Division, The Walter and Eliza Hall Institute for Medical Research, Parkville, VIC 3052, Australia; 5 Department of Medical Biology, The University of Melbourne, Melbourne, VIC 3010, Australia

**Keywords:** Parkin, ubiquitin, ubiquitin ligase, mitophagyĆ, USP30, deubiquitylating enzyme, p97, quantitative proteomics, Ub-clipping

## Abstract

The ubiquitin ligase Parkin, protein kinase PINK1, USP30 deubiquitylase, and p97 segregase function together to regulate turnover of damaged mitochondria via mitophagy, but our mechanistic understanding in neurons is limited. Here, we combine induced neurons (iNeurons) derived from embryonic stem cells with quantitative proteomics to reveal the dynamics and specificity of Parkin-dependent ubiquitylation under endogenous expression conditions. Targets showing elevated ubiquitylation in *USP30*^*−/−*^ iNeurons are concentrated in components of the mitochondrial translocon, and the ubiquitylation kinetics of the vast majority of Parkin targets are unaffected, correlating with a modest kinetic acceleration in accumulation of pS65-Ub and mitophagic flux upon mitochondrial depolarization without USP30. Basally, ubiquitylated translocon import substrates accumulate, suggesting a quality control function for USP30. p97 was dispensable for Parkin ligase activity in iNeurons. This work provides an unprecedented quantitative landscape of the Parkin-modified ubiquitylome in iNeurons and reveals the underlying specificity of central regulatory elements in the pathway.

## Highlights

• Global phospho and ubiquitylome analysis of PINK1-Parkin pathway in iNeurons
• Dynamics and specificity of Parkin-mediated ubiquitylation revealed in iNeurons
• p97-mediated MFN turnover not required for Parkin substrate “gating” in iNeurons
• USP30 acts primarily on translocon and supports import quality control in iNeurons

## Graphical Abstract

**Figure:**
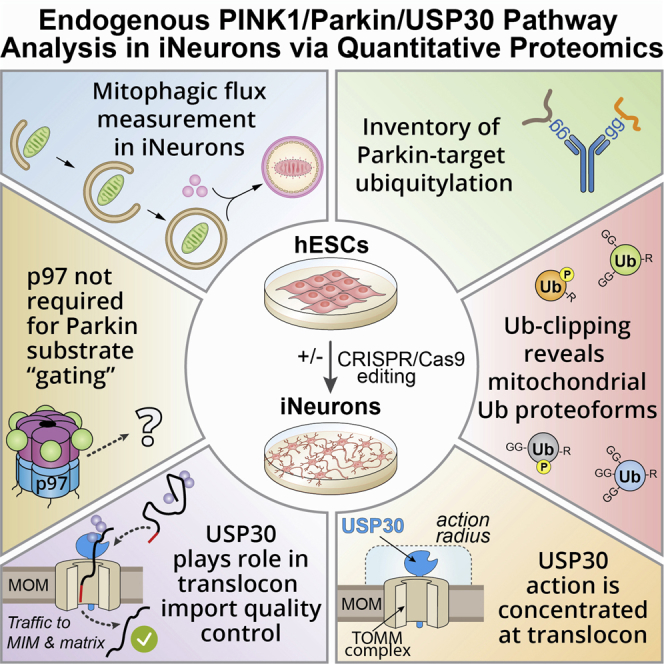

## Teaser

Ordureau et al. combine iNeurons of a desired genotype with multiple quantitative proteomic approaches to define the primary specificity of endogenous Parkin action on mitochondria, the abundance of ubiquitin proteoforms, the role of p97 in remodeling the mitochondrial proteome downstream of Parkin, and the target specificity of USP30 during mitophagic signaling.

## Introduction

Mitochondrial quality control has been associated with various neurodegenerative diseases, including Parkinson’s disease (PD) (Nguyen et al., 2019). Mitochondrial homeostasis is controlled through both biogenesis and removal of damaged mitochondria through a selective form of autophagy called mitophagy. Two genes, *PRKN* and *PINK1*, are mutated in familial early-onset forms of PD and form a surveillance pathway that monitors damaged mitochondria and catalyzes their capture and removal through ubiquitin (Ub)-dependent mitophagy (McWilliams and Muqit, 2017, Truban et al., 2017, Yamano et al., 2016).

*PRKN* encodes the Parkin protein, a E3 Ub ligase that catalyzes Ub transfer upon activation by the PINK1 protein kinase to promote mitophagy (Pickles et al., 2018, Pickrell and Youle, 2015). Our understanding of mechanisms underlying this pathway has been facilitated through analysis of HeLa cells overexpressing Parkin and through structural analysis of Parkin (Gladkova et al., 2018, Harper et al., 2018, Narendra et al., 2008, Sauvé et al., 2018, Wauer et al., 2015). In healthy mitochondria, PINK1 is rapidly imported and degraded (Sekine and Youle, 2018). However, mitochondrial damage, as occurs upon depolarization or accumulation of mis-folded proteins in the matrix (Burman et al., 2017), promotes PINK1 stabilization and activation on the mitochondrial outer membrane (MOM). PINK1 promotes Parkin activation (∼4,400-fold) through a multi-step process involving phosphorylation of pre-existing Ub, recruitment of cytosolic Parkin via its interaction with pS65-Ub on MOM proteins, phosphorylation of S65 in the N-terminal Ub-like (UBL) domain of Parkin by PINK1, and conformational stabilization of Parkin in an active form (Gladkova et al., 2018, Kane et al., 2014, Kazlauskaite et al., 2015, Koyano et al., 2014, Ordureau et al., 2014, Ordureau et al., 2015, Sauvé et al., 2018, Wauer et al., 2015). Parkin retention on the MOM leads to ubiquitylation of a variety of mitochondrial proteins including VDACs, MFNs, RHOTs, and components of the translocon on the MOM (Chan et al., 2011, Geisler et al., 2010, Ordureau et al., 2018, Sarraf et al., 2013). Primary site ubiquitylation is followed by the accumulation of K6, K11, and K63 Ub chains on MOM targets, and ∼20% of Ub molecules on the MOM are phosphorylated on S65 in HeLa cells (Ordureau et al., 2014). The retention of Parkin on the MOM requires this Ub-driven feedforward mechanism involving both increased MOM ubiquitylation and accumulation of pS65-Ub for Parkin binding and activation (Harper et al., 2018, Yamano et al., 2016). Ub chains on mitochondria promote recruitment of Ub-binding autophagy receptors to promote autophagosome assembly and delivery to the lysosome (Heo et al., 2015, Lazarou et al., 2015, Richter et al., 2016, Wong and Holzbaur, 2014).

The MOM-localized deubiquitylating enzyme USP30, which shows selectivity for cleavage of K6-linked Ub chains *in vitro* and in tissue culture cells, has been previously linked with the Parkin pathway (Bingol et al., 2014, Cunningham et al., 2015, Gersch et al., 2017, Marcassa et al., 2018, Sato et al., 2017). Two overlapping models have been proposed. On one hand, overexpression of USP30 can block Parkin-dependent accumulation of Ub chains on MOM proteins in response to depolarization, suggesting that USP30 directly antagonizes Parkin activity (Bingol et al., 2014, Liang et al., 2015, Ordureau et al., 2014). In addition, loss of USP30 can promote the activity of mutant Parkin alleles (Bingol et al., 2014). On the other hand, USP30 has been proposed to associate with the MOM translocon and to control basal ubiquitylation of MOM proteins (Gersch et al., 2017, Marcassa et al., 2018), which is further suggested by the finding that USP30 only poorly hydrolyzes K6-linked Ub chains that are phosphorylated on S65 (Gersch et al., 2017, Sato et al., 2017). Thus, USP30 could control the abundance of pre-existing Ub near the translocon where PINK1 accumulates to set a threshold for Parkin activation. Whether a USP30-driven threshold can be observed experimentally may depend on the strength of the activating signal (i.e., overt depolarization versus endogenous spatially restricted mitochondrial damage) and Parkin levels. Nevertheless, the targets of endogenous USP30 under basal conditions and its role in buffering Parkin activation in neuronal systems are poorly understood.

Given that most mechanistic studies on Parkin involve overexpression systems in HeLa cells, our understanding of Parkin function at endogenous levels and in physiologically relevant cell types is limited. Here, we couple a human embryonic stem cell (hESC) system for production of high-quality induced neurons (iNeurons) of desired genotypes with a suite of unbiased quantitative proteomic approaches to reveal primary ubiquitylation site specificity, ubiquitylation dynamics, Ub phospho-proteoform specificity, protein phosphorylation, and the role of USP30 downstream of endogenous PINK1-Parkin activation. Using diGLY capture proteomics, we quantify the dynamics of ubiquitylation site specificity for dozens of mitochondrial proteins, thereby providing a landscape of endogenous Parkin action in iNeurons. Using “Ub-clipping” by LBPro^∗^ coupled with intact mass analysis (Swatek et al., 2019), we find that PINK1 primarily phosphorylates mono-Ub or the distal Ub in a chain on MOM proteins from depolarized iNeurons. Although the majority of Parkin targets in USP30^−/−^ iNeurons are ubiquitylated on schedule, and Ub phosphorylation is largely unaffected in response to overt mitochondrial depolarization, a small subset of targets, including several subunits of the translocon, are hyper-ubiquitylated during Parkin activation, independently of two additional MOM-associated E3s: MUL1 and MARCH5. However, at sub-threshold levels of depolarization, the absence of USP30 results in a modest acceleration in pS65-Ub accumulation and rate of PINK1-dependent mitophagic flux, as measured using new genetically encoded flux reporters in iNeurons. Importantly, several translocon import substrates that normally travel through the translocon but are not Parkin targets are hyper-ubiquitylated under basal conditions in *USP30*^*−/−*^ iNeurons independently of MUL1 or MARCH5, suggesting a mitochondrial import quality control role for USP30. Finally, in cells overexpressing Parkin, several MOM proteins, including mitofusins (MFN1/2), are ubiquitylated upon depolarization and extracted from the MOM via p97 (also called VCP) for proteasomal delivery (Chan et al., 2011, Tanaka et al., 2010). In this context, removal of MFNs from the MOM has been proposed as a “gating” mechanism that licenses Parkin for subsequent ubiquitylation of other MOM substrates (McLelland et al., 2018). However, the extent to which MOM protein extraction occurs globally as well as the involvement of MFN-dependent gating under endogenous conditions is poorly understood. Surprisingly, we found that MFN1/2 and RHOT1 are the only detectable MOM proteins whose abundance is decreased, albeit <30%, upon depolarization in a p97-dependent manner in the context of endogenous Parkin in iNeurons. Nevertheless, p97 inhibitors do not block primary site ubiquitylation of Parkin substrates in response to depolarization, which is inconsistent with the proposed licensing function for MFN extraction (McLelland et al., 2018). These studies provide a global view of the mitochondrial ubiquitylome dynamics in response to Parkin and PINK1 activation under endogenous protein levels in iNeurons.

## Results

### Development of a Robust Neuronal System for Analysis of Parkin and PINK1-Dependent Signaling

In order to examine Parkin function globally, we sought to develop a robust system for production of iNeurons in larger quantities. hESCs harboring wild-type (WT) Parkin or a homozygous S65A mutation in Parkin’s UBL were subjected to CRISPR-Cas9-based gene editing to homozygously insert an inducible Neurogenin-2 (*NGN2*) cassette into the AAVS1 locus (see STAR Methods; Figure S1A). Mutation of S65A in Parkin is known to decrease mitochondrial recruitment, Ub ligase activity, and the accumulation of pS65-Ub on mitochondria (Kane et al., 2014, Kazlauskaite et al., 2014, McWilliams et al., 2018a, Ordureau et al., 2014, Ordureau et al., 2015, Shiba-Fukushima et al., 2012). In parallel, we created analogous hESCs homozygous for a Parkin^H302A^ allele, which abolishes the binding of Parkin to pS65-Ub (Kazlauskaite et al., 2015, Sauvé et al., 2015, Wauer et al., 2015) (see STAR Methods). NGN2 expression in hESCs produces iNeurons that are known to express markers of excitatory cortical neurons (Zhang et al., 2013, Ho et al., 2016), and upon 10- to 12-day induction, iNeurons were ∼99% positive for the neuronal lineage marker β3-tubulin (Figure S1A). Consistent with iNeurons having a functional Parkin feedforward system and a reliance of pS65-Ub binding to Parkin for initiation of mitochondrial ubiquitylation, we found that iNeurons from cells expressing WT Parkin produced strong depolarization-dependent accumulation of pS65-Ub and ubiquitylated MFN2, but these events were absent in Parkin^H302A^ iNeurons (∼99% β3-tubulin positive) (Figure S1B). Moreover, Parkin phosphorylation on S65 within its UBL domain was robust in iNeurons with WT Parkin but not detected with the Parkin^H302A^ mutant (Figure S1B), consistent with a model wherein unphosphorylated Parkin binds to pre-existing Ub on the MOM that has been phosphorylated by PINK1, leading to subsequent phosphorylation of Parkin’s N-terminal UBL-domain on S65 by PINK1 (Harper et al., 2018, Sauvé et al., 2015, Wauer et al., 2015).

**Figure 1:**
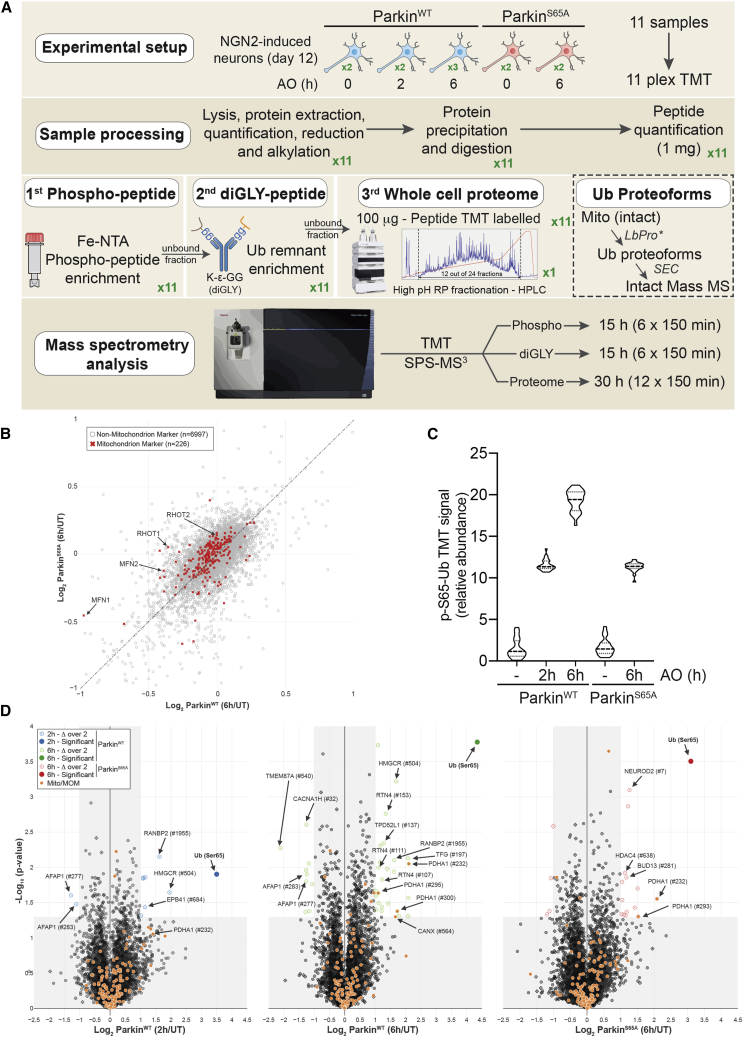
Global Proteomic and Phospho-Proteomic Analysis of iNeurons in Response to Parkin Activation (A) 11-plex TMT-MS^3^-based proteomic analysis of iNeurons to examine total, phospho, Ub proteoforms, and Ub-modified proteomes. (B) Correlation plot for mitochondrial protein abundance for WT or S65A-Parkin iNeurons as determined by log_2_ ratio in abundance comparing untreated cells versus cells that were depolarized for 6 h. (C) Relative abundance of pS65-Ub peptides in WT or S65A-Parkin iNeurons with or without depolarization with AO for 2 or 6 h. Values based on 24 individual MS^3^ analyses for the pS65-Ub tryptic peptide. (D) Volcano plots for phosphoproteomes of WT and S65A-Parkin iNeurons depolarized for 2 or 6 h compared with untreated cells. Phospho-peptide abundance was normalized against the total protein abundance when available (circle) or not (square). Phospho-peptides with log_2_ ratio < −1 or > 1 (p < 0.05) are indicated as colored empty dotted circles/squares, and filled colored circles/squares indicate statistically significant hits (Welch’s t test [S0 = 1], corrected for multiple comparison by permutation-based false discovery rate [FDR] [5%]). The phosphorylated residue is indicated after the pound sign. Phospho-peptide of proteins associated with mitochondria (MitoCarta 2.0 [Calvo et al., 2016] or mitochondrial outer membrane identified by proximity biotinylation [Hung et al., 2017]) are indicated in orange. See also Figure S1 and Table S1.

Using this iNeuron system, we developed a tandem mass tagging (TMT)-MS^3^-based pipeline (Figure 1A) to quantify total proteome abundance, total cellular phosphoproteome, mitochondrial phospho-Ub proteoforms, and total cellular ubiquitylome upon mitochondrial depolarization (McAlister et al., 2014, Rose et al., 2016). This system allowed us to compare duplicate or triplicate cultures of WT or S65A-Parkin iNeurons that were either untreated or depolarized with 10 μM antimycin A/5 μM oligomycin A (AO) for 2 or 6 h in a single experiment (Figure 1A). We found that depolarization for 2 or 6 h resulted in very small changes in proteome abundance, with the abundance of <6 proteins of the 7,223 proteins quantified (Table S1) significantly reduced and <5 proteins significantly increased after AO (log_2_ ratio < −1.0 or > 1.0, log_10_ p value > 1.5), regardless of the genotype or time of AO treatment (Figures S1C and S1D). However, we did note a trend in the reduction of a cohort of mitochondrial proteins that was dependent on the presence of WT Parkin and that included MFNs and RHOTs (Figure 1B). Moreover, among several organellar proteomes, mitochondrion was the only one for which the mean extent of reduction 6 h post-depolarization was statistically distinct from untreated cells (Figure S1E).

### Global Phosphoproteome Analysis during Parkin-Dependent Mitophagy in iNeurons

In response to depolarization, PINK1 is stabilized on the MOM, where it has been proposed to phosphorylate both pre-existing Ub and Ub chains assembled by Parkin, as well as several additional proteins on the MOM (MFNs, RHOTs, RAB8/8A, and Rab13) (Chen and Dorn, 2013, Kane et al., 2014, Koyano et al., 2014, Lai et al., 2015, Ordureau et al., 2018, Shlevkov et al., 2016). We therefore examined global phosphorylation using 11-plex TMT-MS^3^ in response to depolarization in iNeurons expressing WT or S65A-Parkin with or without depolarization (2 or 6 h) (Figure 1A; Table S1). From >8,000 phospho-peptides identified and quantified in cells with WT Parkin, only a single phospho-peptide, corresponding to pS65-Ub, was found to be significant, with a log_2_ ratio > 2.0 and a −log_10_ p value greater than 1.3 (Figures 1C and 1D). Consistent with previous experiments (Ordureau et al., 2018), Parkin^S65A^-expressing cells also accumulated pS65-Ub 6 h post-depolarization, to an extent comparable with that seen at 2 h post-depolarization with WT Parkin (Figures 1C and 1D). However, this analysis did not identify other previously reported PINK1 substrates. We note that the properties of the tryptic peptide corresponding to pS65 in Parkin make it challenging for routine detection by proteomics. Taken together, these data suggest that Ub is a major target of PINK1, independent of Parkin phosphorylation on its UBL.

### Inventory of pS65-Ub Proteoforms Generated during Parkin Action in iNeurons

**Figure 2:**
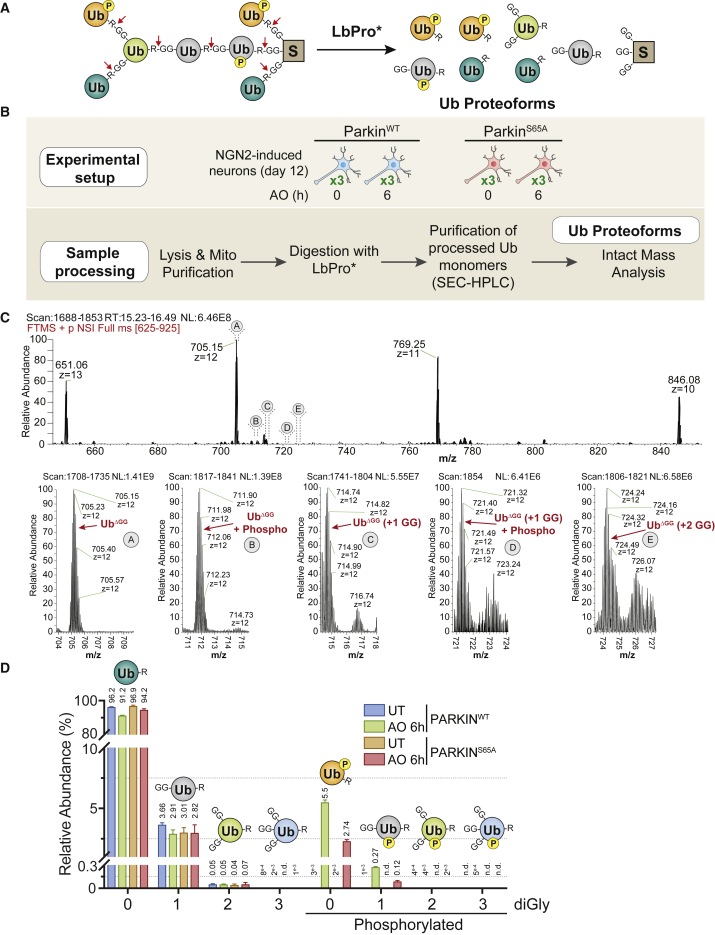
Quantitative Analysis of pS65-Ub Proteoforms on the MOM in iNeurons using Ub Clipping and Intact Mass Spectrometry (A) Scheme depicting the products produced by LbPro^∗^ on distinct Ub proteoforms. (B) Workflow for analysis Ub proteoforms in iNeurons. Mitochondria were purified from iNeurons expressing WT or S65A-Parkin with or without depolarization (6 h). Mitochondrial extracts were treated with LbPro^∗^ and Ub monomers purified by size exclusion chromatography. Samples were then subjected to LC-intact mass analysis prior to quantification of isotopic clusters for phospho and diGLY-containing Ub^ΔGG^ proteoforms across all charge state distributions. (C) Mass spectra for individual Ub^ΔGG^ proteoforms in response to depolarization of WT iNeurons. Shown below are spectra of the isotopic cluster for the z = 12 precursors. (D) Relative abundance of Ub^ΔGG^ proteoforms. Error bars represent SEM; n = 3. n.d., not determined. See also Figure S2.

Given that Ub phosphorylation and Parkin-dependent MOM protein ubiquitylation are integrated to generate mitophagy signals (Harper et al., 2018, Pickrell and Youle, 2015, Yamano et al., 2016), we next sought to understand the complexity of phosphorylated Ub proteoforms during mitophagic signaling in iNeurons. We used LbPro^∗^, a protease that specifically cleaves Ub after R74 to produce Ub^ΔGG^, thereby leaving a diGLY peptide linked with lysine residues on primary ubiquitylation sites and the Ub molecule itself (corresponding to chain extension at one of seven lysines in Ub) (Figure 2A) (Swatek et al., 2019). Ub^ΔGG^ molecules released from cellular proteins by LbPro^∗^-mediated “clipping” can be analyzed using intact liquid chromatography-mass spectrometry (LC-MS) to quantify the ensemble of Ub proteoforms, including the degree of chain branching and additional modification states such as phosphorylation (Figure 2A). To facilitate analysis, we developed a method for purification of Ub^ΔGG^ monomers, produced by LbPro^∗^ of enriched mitochondria from cells with or without depolarization using size exclusion chromatography (SEC) (Figure 2B). To validate the method, we first performed the workflow on mitochondria from HeLa cells expressing inducible Parkin, which allowed us to demonstrate that (1) Ub phosphorylation delays elution during LC-MS, and (2) the charge state distributions of Ub^ΔGG^ and pS65-Ub^ΔGG^ are distinct (Figures S2A and S2B). Thus, quantification of Ub^ΔGG^ proteoforms requires integration of all Ub species and deconvolution of charge states (see STAR Methods), which ultimately allowed us to confidently detect and quantify Ub^ΔGG^ molecules containing up to three diGLYs with or without phosphorylation (Figures 2A and S2C). In the HeLa cell system, 12% of Ub^ΔGG^ was present as a phosphorylated but unbranched proteoform after 1 h of depolarization (Figure S2D), comparable with that found previously with targeted proteomics system (Ordureau et al., 2014). Much smaller amounts of Ub^ΔGG^ (1.42% and 0.05%) were present as phosphorylated proteoforms with one or two diGLY isopeptide branches present, respectively (Figure S2D). Phosphorylated Ub^ΔGG^ proteoforms were not detected in cells that were not depolarized, consistent with PINK1 activation’s being essential for production of pS65-Ub (Figure S2D). Overall, these data fit well with an independent analysis for LbPro^∗^-based Ub proteoform analysis in HeLa cells (Swatek et al., 2019).

We then purified mitochondria from iNeurons expressing WT or S65A-Parkin with or without depolarization for 6 h and performed the LbPro^∗^ workflow (Figure 2B). In total, 5.5% and 0.27% of Ub^ΔGG^ was present in a phosphorylated form with no or a single diGLY branch, respectively, after depolarization (Figures 2C and 2D). These species were undetectable in the absence of depolarization and were reduced in abundance by ∼2-fold in iNeurons expressing Parkin^S65A^, consistent with previous studies (Ordureau et al., 2018). Much smaller levels of Ub containing two or three diGLY proteoforms were detected (Figures 2C and 2D). Thus, the majority of Ub phosphorylation in iNeurons in response to depolarization occurs on monomeric Ub or on the distal Ub molecule within a chain. This is also in line with the finding that PINK1 preferentially phosphorylates the distal Ub molecule within a K6 or K11 chain *in vitro* (Gersch et al., 2017, Sato et al., 2017).

### Quantitative diGLY Proteomics in iNeurons Reveals Global Landscape of Parkin Action

**Figure 3:**
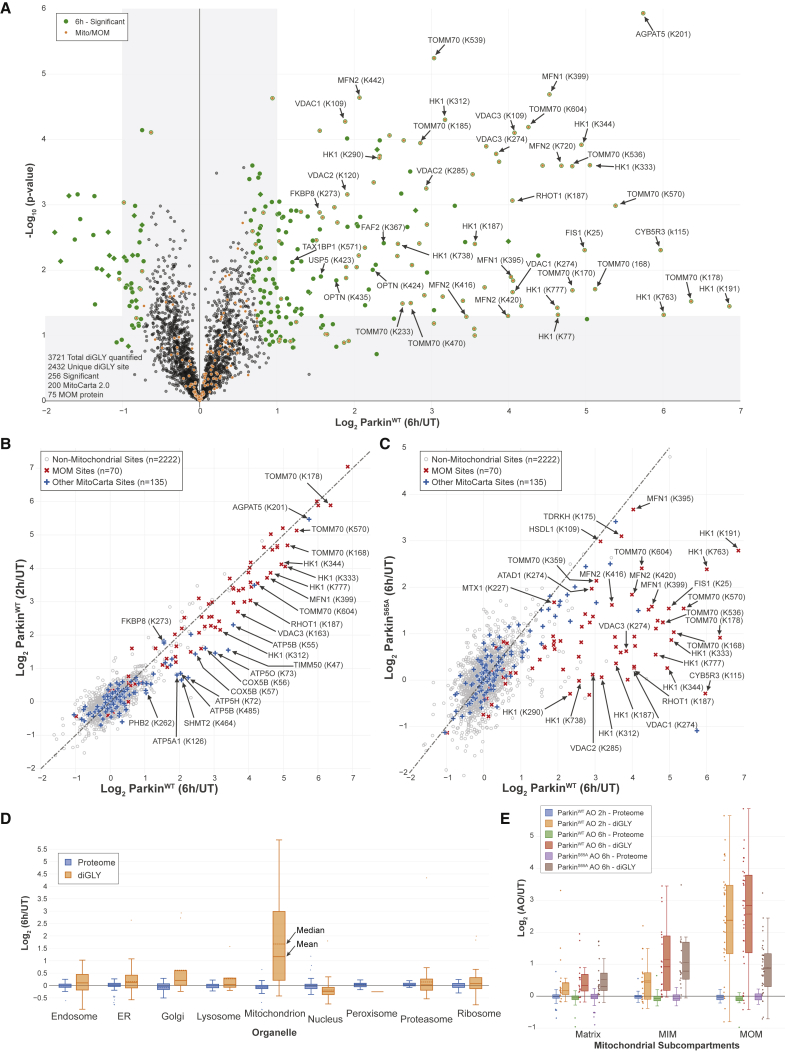
Quantitative Discovery of the Parkin-Dependent Ubiquitylome in iNeurons during Mitophagic Signaling (A) Volcano plot of diGLY-containing peptides identified by TMT-MS^3^ with or without depolarization of iNeurons for 6 h. diGLY-peptide abundance was normalized against total protein abundance when available (circle) or not (square). Filled colored circles/squares indicate statistically significant hits (Welch’s t test [S0 = 1], corrected for multiple comparison by permutation-based FDR [1%]). diGLY-modified residue is indicated in bracket after the protein name. diGLY-peptide of proteins associated with mitochondria (MitoCarta 2.0 [Calvo et al., 2016] or mitochondrial outer membrane identified by proximity biotinylation [Hung et al., 2017]) are overlaid with an orange circle. (B) Correlation plot of log_2_(AO/UT) for WT iNeuron diGLY sites after depolarization for 2 h (y axis) or 6 h (x axis). (C) Correlation plot of log_2_(AO/UT) for diGLY sites from WT (y axis) or Parkin^S65A^ (x axis) iNeurons after depolarization for 6 h. (D) Distribution of changes in protein abundance (blue) or diGLY peptides (orange) for proteins that localize in individual organelles or protein complexes. (E) Distribution of changes in protein abundance or diGLY peptides for proteins that localize in the mitochondria matrix, the MIM, or the MOM. See also Figure S3 and Table S1.

Having examined global proteome, phosphoproteome, and phospho-Ub proteoform alterations in iNeurons in response to mitochondrial depolarization, we then examined the cellular ubiquitylome and the role of Parkin phosphorylation on S65 using diGLY affinity capture coupled with quantitative proteomics (Figure 1A) (Rose et al., 2016). Tryptic peptides from whole-cell extracts of iNeurons containing WT or S65A-Parkin without or with depolarization (2 or 6 h) were subjected to α-diGLY immunoprecipitation and samples analyzed using 11-plex TMT-MS^3^, with diGLY peptide intensities normalized with total protein abundance measured in parallel (Figures 3A–3C and S3A–S3D; Table S1) (see STAR Methods). From more than 2,400 unique diGLY-containing peptides quantified, we identified 134 ubiquitylation sites in 83 proteins at 6 h whose abundance was increased by >2-fold (p < 0.05) (Figure 3A). Most of these sites (101) were found at 2 h of depolarization, with an additional 32 sites displaying log_2_ ratios > 1.0 only at 6 h (Figures 3A, 3B, and S3A). Only 8 sites were identified as changing at 2 h but not at 6 h. In contrast, depolarization of Parkin^S65A^ iNeurons for 6 h resulted in a reduced ubiquitylation signature, consistent with lower activity in the absence of UBL phosphorylation (Figures 3C, S3B, and S3C). As expected, principal-component analysis revealed that the 2 and 6 h depolarization data were more similar to each other than to the untreated or Parkin^S65A^ samples, which were also more similar to each other (Figure S3D).

The most dramatic shift in diGLY peptide abundance compared with the total proteome for individual organelles (Itzhak et al., 2016) was found for mitochondrial proteins (Figure 3D). Indeed, 80% of Parkin substrates targeted after 2 h of depolarization are MOM localized, and the majority of these proteins and sites were also observed 6 h post-depolarization (Figures 3B and 3C). We observed several mitochondrial inner membrane (MIM) or matrix proteins that have increased abundance of diGLY sites after 6 h of depolarization (Figure 3E). This may reflect the fragmentation of mitochondria that is known to occur after mitochondrial damage (Yoshii et al., 2011), although we cannot exclude the alternative explanation wherein abundant MIM or matrix proteins are stalled on the translocon, where they may be ubiquitylated by Parkin (Sarraf et al., 2013).

### Quantitative Analysis of p97-Dependent MOM Protein Turnover during Parkin-Dependent Mitophagy

**Figure 4:**
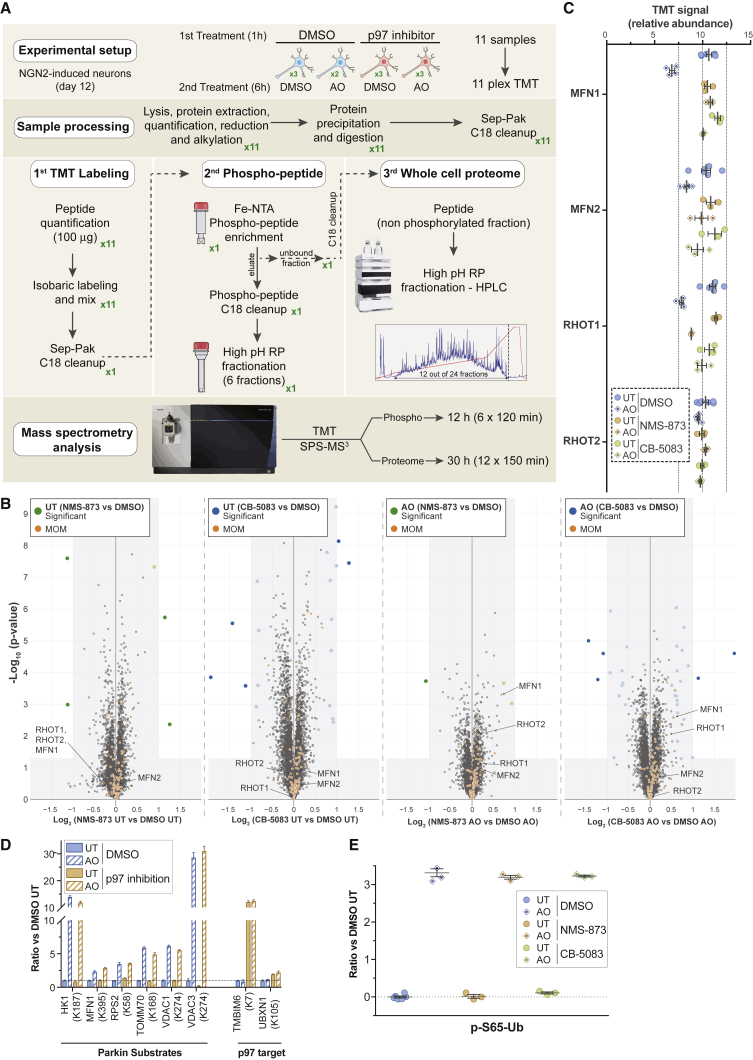
Quantitative Analysis of p97-Dependent Mitochondrial Proteome Remodeling and PINK1-Parkin Pathway Activity in iNeurons (A) 11-plex TMT-MS^3^-based proteomic analysis of iNeurons to examine total, phospho, and Ub-modified proteomes with or without inhibition of p97. (B) Volcano plots for total proteomes of iNeurons either left untreated or depolarized for 6 h in the presence of absence of the p97 inhibitors NMS-873 or CB-5083. Only proteins quantified in both p97 inhibitor dataset and also quantified with more than one peptide are represented (circle). Filled colored circles/squares indicate statistically significant hits (Welch’s t test [S0 = 0.585], corrected for multiple comparison by permutation-based FDR [1%]). MOM proteins identified by proximity biotinylation (Hung et al., 2017) are overlaid with an orange circle. (C) Relative abundance of MFN1/2 and RHOT1/2 in iNeurons either left untreated or depolarized for 6 h in the presence or absence of the p97 inhibitors NMS-873 or CB-5083. Error bars represent SEM. (D) Relative abundance (measured as ratio in the presence of AO versus untreated) of individual diGLY-containing peptides from the indicated Parkin substrates or p97 targets in iNeurons either left untreated or depolarized for 6 h in the presence of absence of the p97 inhibitor. Error bars represent SEM. (E) Relative abundance (measured as ratio in the presence of AO versus untreated) of pS65-Ub in iNeurons either left untreated or depolarized for 6 h in the presence or absence of the p97 inhibitors NMS-873 or CB-5083. Error bars represent SEM. See also Figure S4 and Table S2.

We next used the iNeuron system to examine additional regulatory components in the pathway. Previous studies largely in HeLa cells with overexpressed Parkin demonstrated that several MOM proteins, including MFNs, are rapidly ubiquitylated and degraded by the proteasome (Chan et al., 2011, McLelland et al., 2018, Tanaka et al., 2010). This process is thought to require extraction of the ubiquitylated proteins from the membrane via the p97 AAA ATPase (McLelland et al., 2018, Tanaka et al., 2010). However, the extent of this process with endogenous Parkin levels and the breadth of MOM proteins targeted in this way is poorly understood. To examine global and mitochondrion-specific changes in protein abundance in iNeurons upon depolarization with or without addition of p97 inhibitors, we performed 11-plex TMT-MS^3^ on total cell extracts (Figure 4A; Table S2). We used two p97 inhibitors: CB-5083, an ATP-competitive inhibitor (Anderson et al., 2015), and NMS-873, an ATP-non-competitive inhibitor (Magnaghi et al., 2013). Volcano plots comparing non-depolarized and depolarized (6 h) cells with or without either of the two inhibitors revealed only minor alterations in proteome abundance, with fewer than 3 proteins increasing or decreasing in abundance in the presence of either of the p97 inhibitors for >5,600 proteins quantified across both datasets (Figures 4B, S4A, and S4B). Principal-component analysis revealed that 31% of changes in proteome abundance are a result of depolarization (component 1), while 17% of the changes reflect the p97 inhibitors (component 2, primarily the more potent inhibitor CB-5083) (Figure S4B). Consistent with p97 inhibition, the abundance of K6, K11, K33, and K48 linkages in Ub were all increased 2- to 5-fold, regardless of depolarization (Figure S4C) (Anderson et al., 2015, Heidelberger et al., 2018, Magnaghi et al., 2013). Remarkably, the only MOM proteins whose abundance was decreased by depolarization in a p97-dependent manner were MFN1 and RHOT1 (log_2_ ratio ∼ −0.5, p > 0.05), although the abundance was decreased by only 30% and 20%, respectively (Figures 4B, 4C, and S4A). MFN2 abundance was also reduced by ∼20%, although the p value did not reach significance, while RHOT2 was not detectably reduced (Figures 4B, 4C, and S4A). The small reductions in MFN1 and RHOT1 levels observed here at endogenous Parkin levels are much less than observed with Parkin overexpression, in which MFN levels are often greatly reduced or eliminated at shorter times post-depolarization (McLelland et al., 2018, Tanaka et al., 2010). These data suggest that p97 does not re-sculpt the MOM is a dramatic way.

### MFN Turnover via p97 Is Not Required for Parkin Pathway Activation in iNeurons

Prior studies have proposed a “gating” mechanism for Parkin-dependent ubiquitylation of proteins on the MOM (McLelland et al., 2018). Here, ubiquitylation of MFN by Parkin promotes MFN extraction from the MOM and degradation by the proteasome, and only after MFN degradation is Parkin licensed to ubiquitylate other MOM proteins. The loss of MFN from the MOM is proposed to release mitochondrial-ER contact sites to promote Parkin substrate licensing (McLelland et al., 2018). This model makes two strong predictions: First, inhibition of MFN extraction from the MOM via p97 inhibitors should block Parkin-dependent primary ubiquitylation of other MOM substrates. Second, p97 inhibition should block the accumulation of pS65-Ub that is dependent upon Parkin “licensing” and activation of the feedforward process, as the absence of Parkin activity on bulk MOM substrates would reduce the availability of Ub chains for PINK1-dependent phosphorylation. However, in contrast to expectations based on the “gating” model, ubiquitylation of HK1, MFN1, TOMM70, VDAC1, and VDAC3 was unchanged with p97 inhibition (Figure 4D; Table S2). Second, we performed phosphoproteome enrichment on trypsinized iNeuron proteins (Figures 4A, S4D, and 4E; Table S2), allowing a quantitative analysis of pS65-Ub abundance in the absence of p97 activity. pS65-Ub levels were unchanged in the presence of p97 inhibition (Figure 4E), despite inhibition of MFN1 and RHOT1 turnover (Figure 4C). Taken together, we conclude that MFN extraction/degradation is not a pre-requisite for Parkin-dependent ubiquitylation of a variety of MOM proteins and for activation of the feedforward mechanism in iNeurons.

### Role of USP30 in Parkin-Dependent Mitochondrial Ubiquitylation in iNeurons

**Figure 5:**
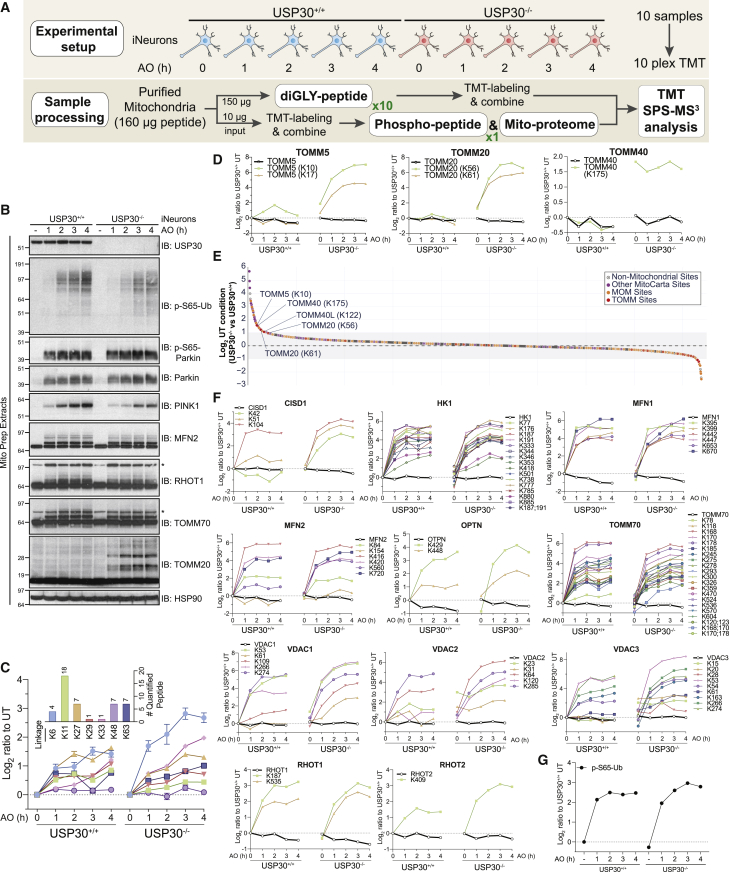
Selectivity of USP30-Dependent Buffering of MOM Ubiquitylation in iNeurons with and without Depolarization (A) Workflow for analysis of USP30-dependent mitochondrial ubiquitylation. (B) WT or *USP30*^*−/−*^ iNeurons were left untreated or depolarized with 10 μM antimycin A/5 μM oligomycin A as indicated and mitochondria immunoblotted with the indicated antibodies. (C) Time course for quantified Ub chain linkage types in iNeurons with or without USP30 in response to depolarization as in (B). TMT intensities were normalized to untreated separately for each genotype. The inset shows the number of peptides used for quantification. Error bars represent SEM. (D) Ubiquitylation kinetics for individual diGLY sites (and protein level in black) in TOMM5, TOMM20, and TOMM40 for WT and *USP30*^*−/−*^ iNeurons in response to depolarization as in (B). (E) Log_2_ ratio under untreated conditions of diGLY peptide intensities for *USP30*^*−/−*^ versus WT cells. Legend displays the color code for mitochondrial and non-mitochondrial diGLY sites. (F) Ubiquitylation kinetics for individual diGLY sites (and corresponding protein level in black) in MOM-localized Parkin targets for WT and *USP30*^*−/−*^ iNeurons in response to depolarization as in (B). (G) Time course for Ub phosphorylation on S65 in iNeurons with or without USP30 in response to depolarization as in (B). See also Figure S5 and Table S3.

Mitochondrially localized USP30 is considered to be an important negative regulator of mitophagy (Bingol et al., 2014, Cunningham et al., 2015, Gersch et al., 2017, Liang et al., 2015, Marcassa et al., 2018, Sato et al., 2017). Previous studies demonstrated that USP30 can reverse ubiquitylation of a subset of Parkin substrates or completely remove ubiquitin chains (Bingol et al., 2014, Cunningham et al., 2015, Gersch et al., 2017, Ordureau et al., 2014, Sato et al., 2017). Data derived from cell lines depleted of USP30 have led to a model wherein USP30 functions in Parkin-independent basal mitophagy and pexophagy by continually downregulating basal ubiquitination of these organelles (Marcassa et al., 2018). Mechanistically, USP30 comprises two Ub binding sites that furnish it with preference for Lys6-linked polyUb (Gersch et al., 2017, Sato et al., 2017). However, whether USP30 also comprises specificity at the level of primary ubiquitylation sites in MOM substrates, and the extent to which USP30 activity suppresses the Parkin feedforward activation mechanism via pS65-Ub, has not been examined in more physiological systems such as neurons. Therefore, we constructed *USP30*^*−/−*^ hESCs containing AAVS1-NGN2 and converted parental WT and *USP30*^*−/−*^ cells to iNeurons (>97.5% on the basis of β3-tubulin staining) (Figures 5A, 5B, and S5A). We then either left cells untreated or subjected cells to overt depolarization for 1, 2, 3, or 4 h with 10 μM antimycin A/5 μM oligomycin A prior to isolation of mitochondria (Figure 5A) (see STAR Methods). Mitochondria were then trypsinized and processed for total proteome analysis or phosphoproteome analysis or subjected to the diGLY enrichment workflow. Samples were subjected to 10-plex TMT-MS^3^ analysis, and the abundance of each diGLY and phospho modification was normalized to the abundance of the total protein at each time point (Figures 5C–5F, S5B, S5C, S6A, S6B, and S6D; Table S3).

Analysis of these data led to several findings. First, we observed enhanced accumulation of multiple chain linkage types upon depolarization in *USP30*^*−/−*^ cells relative to *USP30*^*+/+*^ cells (Figure 5C). The largest fold increase was seen with K6 linkages, consistent with the finding that USP30 has a preference for cleavage of K6 diUb linkages *in vitro*, although it also cleaves K11, K27, K48, and K63 linkages at lower rates *in vitro* (Cunningham et al., 2015, Gersch et al., 2017, Sato et al., 2017).

Second, a cohort of individual ubiquitylation sites on a small set of Parkin targets displayed little to no ubiquitylation over the 4 h time course in the presence of USP30, but were dramatically ubiquitylated in *USP30*^*−/−*^ cells (Figures 5D, S5B, and S5C). This included K10 and K17 in TOMM5, as well as K56 and K61 in TOMM20, and more slowly migrating ubiquitylated forms of TOMM20 were confirmed by immunoblotting in *USP30*^*−/−*^ cells (Figure 5B).

Third, under basal conditions, we identified two classes of ubiquitylation events that were 1.5-fold or more abundant in *USP30*^*−/−*^ cells than in WT cells (Figures 5E and 5F): (1) MOM protein ubiquitylation that was elevated under basal conditions and did not change in abundance during depolarization, including TOMM40-K175, TOMM40L-K122, and several events in the N-terminal helical domain of VDAC2 and VDAC3; and (2) MOM protein ubiquitylation that was elevated under basal conditions and increased in abundance during depolarization, including individual sites in HK2, FIS1, VDAC1, CYB5R3, TOMM20, and TOMM5.

Fourth, a large set of MOM-localized Parkin targets displayed similar rates of accumulation of ubiquitylation with or without USP30 for many or all of the individual diGLY sites detected, with the vast majority of sites not being elevated in *USP30*^*−/−*^ cells under basal conditions (Figure 5F). Examples include the majority of ubiquitylation events in HK2 and TOMM70, as well as RHOT1 and MFN2, with major ubiquitylated forms validated by immunoblotting (Figure 5B). Of note, modification of K42 and K51 (but not K104) in CISD1 was elevated in *USP30*^*−/−*^ iNeurons during depolarization, as was K78 (but not a large number of other sites) in TOMM70, suggesting that some substrates display selectivity in the response to USP30 loss.

Finally, in global phospho-proteomic analysis of mitochondria, we found that loss of USP30 had no obvious effect on the abundance of pS65-Ub under basal conditions or on the rate of pS65-Ub accumulation in response to overt depolarization (Figures 5G, S6A, and S6B), a result that was confirmed by immunoblotting of mitochondrial proteins with α-pS65-Ub antibody (Figure 5B). USP30 deletion also had no obvious effect on the abundance of pS65-Parkin, although PINK1 abundance was slightly decreased relative to *USP30*^*+/+*^ cells (Figure 5B). In this experiment, we found that pS65 in Ub was present on an otherwise unmodified peptide as well as the peptide containing diGLY on K63 (Figure S6A), as seen previously in HeLa cells (Ordureau et al., 2014). No other phospho-peptides were identified on purified mitochondria whose abundance was significantly altered by the absence of USP30 in this setting (Figure S6A; Table S3).

### USP30 Modestly Restrains Mitophagic Flux and Ub Phosphorylation with Sub-threshold Depolarization

**Figure 6:**
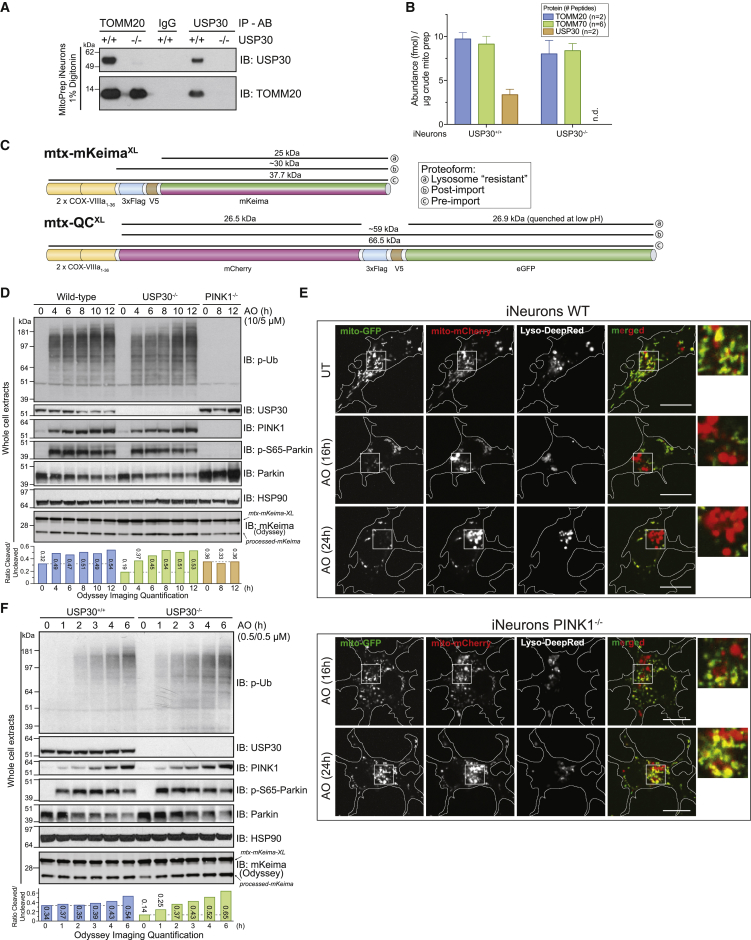
USP30 Modestly Restrains Mitophagic Flux and Ub Phosphorylation with Sub-threshold Depolarization in iNeurons (A) *USP30*^*+/+*^ or *USP30*^*−/−*^ iNeurons were lysed and extracts subjected to immunoprecipitation using α-TOMM20, α-USP30, or control α-IgG antibody prior to analysis by SDS-PAGE and immunoblotting. (B) The absolute abundance of TOMM20, TOMM70, and USP30 in purified mitochondria was measured using parallel reaction monitoring using two, six, and two heavy-reference tryptic peptides. The average number of fmol of each protein/μg of mitochondria is shown. Error bars represent SEM for peptide measurements. (C) Schematic of the flux reporter constructs. mtx-Keima^XL^ contains dual mitochondrial targeting sequences from COXVIII, a FLAG-V5 epitope, and the Keima protein. The sizes of full-length, post-import, and lysosomally processed (“resistant”) proteins are indicated. mtx-QC^XL^ contains dual mitochondrial targeting sequences from COXVIII, mCherry, a FLAG-V5 epitope, and GFP. The size of the full-length protein as well as the products of cleavage in the lysosome are shown. (D) The indicated iNeurons were depolarized with 10 μM antimycin A/5 μM oligomycin (overt depolarization) for the indicated times and cell extracts immunoblotted with the indicated antibodies. The relative ratios of processed to unprocessed Keima were measured using quantitative immunoblotting with Odyssey (see STAR Methods). (E) WT or *PINK1*^*−/−*^ iNeurons expressing mtx-QC^XL^ were depolarized as in (F) and imaged for mCherry, GFP, and Lysotracker Deep-Red at the indicated time points. Cells were imaged as described in STAR Methods. (F) The indicated iNeurons were depolarized with 0.5 μM antimycin A/0.5 μM oligomycin (sub-threshold depolarization) for the indicated times and cell extracts examined as in (D). See also Figure S6 and Table S4.

Given previous models concerning USP30 function, the finding that pS65-Ub accumulation was indistinguishable in *USP30*^*+/+*^ and *USP30*^*−/−*^ cells with overt depolarization was surprising. PINK1 is known to accumulate on the translocon, and increased ubiquitylation near the translocon could increase the local Ub concentration to support its phosphorylation by PINK1 and subsequent recruitment and activation of Parkin. Moreover, we found that USP30 associates with TOMM20 in iNeurons on the basis of co-immunoprecipitation analysis (Figure 6A), as previously found in HeLa cells (Liang et al., 2015). Using parallel reaction monitoring-based proteomics, we found that the stoichiometry of USP30 to TOMM20 and TOMM70 was ∼0.3 in purified mitochondria, indicating that a substantial fraction of translocon assemblies are likely to have associated USP30 (Figure 6B). One potential explanation for no effect on the feedforward process with overt depolarization is that activated Parkin rapidly inactivates USP30 or otherwise outpaces Ub removal by USP30, as previously proposed (Bingol et al., 2014, Cunningham et al., 2015). We therefore reasoned that treating cells with lower levels of AO (referred to here as “sub-threshold”) might reveal alterations in the kinetics of pS65-Ub accumulation or mitophagic flux. However, the extent to which neuronal cells undergo Parkin and PINK1-dependent mitophagic flux is controversial, in part because of the finding that *PINK1*^*−/−*^ mice do not have obvious defects in mitophagic flux and also because most mitophagy studies in cultured neurons have not used flux reporters (Ashrafi et al., 2014, Cai et al., 2012, McWilliams et al., 2018a, McWilliams et al., 2018b). Therefore, to address PINK1 and USP30-dependent mitophagic flux in iNeurons, we developed two new mitophagic flux reporters (Figure 6C) that build upon prior work (Allen et al., 2013, An and Harper, 2018, Katayama et al., 2011). mtx-Keima^XL^ is a matrix-targeted reporter of mitophagic flux that undergoes a red shift in excitation maxima upon reduction in pH within the lysosome (Katayama et al., 2011), allowing mitophagic flux to be examined using live-cell imaging. We also included a FLAG-V5 epitope whose cleavage within the lysosome leads to the production of a 25 kDa processed Keima protein that is resistant to further degradation, allowing mitophagic flux to be independently measured by ratio of post-imported mtx-Keima^XL^ to lysosome resistant processed form of Keima (which we will refer to as “processed” Keima; Figure 6C) (An and Harper, 2018). In addition, we created mtx-QC^XL^, a matrix-targeted mCherry-GFP protein that undergoes quenching of the GFP moiety upon acidification in the lysosome but retains mCherry fluorescence, allowing flux to be examined microscopically (Figure 6C) (Allen et al., 2013). As with mtx-Keima^XL^, we also included a FLAG-V5 epitope between the mCherry and GFP sequence whose cleavage within the lysosome leads to the production of a 26.5 kDa processed mCherry and 26.9 kDa processed eGFP protein that are resistant to further degradation, allowing measurement of flux by quantitative immunoblotting. These constructs were introduced into engineered hESCs using PiggyBac vectors.

We verified that iNeurons expressing mtx-Keima^XL^ maintain the expected properties with overt depolarization (Figure 6D). At 4 h post-depolarization, pS65-Ub accumulation was similar in both *USP30*^*+/+*^ and *USP30*^*−/−*^ cells and, as expected, was completely absent in *PINK1*^*−/−*^ iNeurons (Figure 6D). WT iNeurons display substantial PINK1-independent basal flux, including a ratio of processed to unprocessed Keima of ∼0.3 and the presence of several mCherry-positive puncta in mtx-QC^XL^-expressing cells (Figures 6D, 6E, S6C, and S6D). In WT cells undergoing overt depolarization, Keima processing increased from ∼0.3 to ∼0.5 over the 4- to 12 h period, and this increase was PINK1 dependent, consistent with an increase in flux occurring through the PINK1-Parkin pathway (Figure 6D). Although *USP30*^*−/−*^ cells had a lower rate of basal flux (∼0.2), flux was also near maximal (∼0.4–0.5) within 4 h of overt depolarization.

We then examined pS65-Ub accumulation and mitophagic flux at sub-threshold AO. Unlike overt depolarization, *USP30*^*−/−*^ cells displayed modest acceleration of pS65-Ub accumulation at the earliest time point, with prominent phosphorylation seen at 1 h post-depolarization but delayed to 2 h in *USP30*^*+/+*^ cells (Figure 6F). Moreover, although the basal flux was slightly lower in *USP30*^*−/−*^ cells than WT cells (∼0.2 versus ∼0.3), the increase in flux at sub-threshold depolarization was modestly accelerated in *USP30*^*−/−*^ cells relative to WT cells (Figure 6F). As expected, increased flux as measured with mtx-QC^XL^ in live cells with sub-threshold depolarization was also PINK1 dependent (Figure S6D). These data indicate that although USP30 removal from cells has a modest effect on the kinetics of pS65-Ub accumulation and mitophagic flux at sub-threshold depolarization, cells with endogenous Parkin can readily overcome this delay to allow maximal activation of the Parkin feedforward ubiquitylation pathway. This is in line with a model proposing that USP30 inhibition would be helpful to boost mitophagy flux, by lowering the threshold of damage needed to initiate Parkin activation and the feedforward system. These data also demonstrate that in addition to basal mitophagic flux, PINK1/Parkin-dependent flux can be measured in post-mitotic neurons.

### Mitochondrial Matrix Targeted Proteins Are Ubiquitylated Basally in USP30^−/−^ iNeurons

Among the proteins whose ubiquitylation was increased basally on mitochondria from USP30^−/−^ iNeurons were several proteins that are imported into mitochondria via the translocon, including citrate synthase (CS), several subunits of complex I, complex IV, and complex V (ATP synthase) (Figures S6E and S6F). However, unlike translocon subunits such as TOMM20, these proteins were not further ubiquitylated upon Parkin activation, suggesting that they are targeted for ubiquitylation through an alternative mechanism. Interestingly, the majority of ubiquitylation sites are located near the C terminus of the target protein (Figure S6E). We validated that CS is ubiquitylated under basal conditions in the absence of USP30 but not in its presence using immunoblotting of proteins enriched for ubiquitylated proteins, and this was not affected by overt depolarization (4 h) (Figure S7A). However, the abundance of ubiquitylated forms of TOMM20 or CS was largely unchanged in *USP30*^*−/−*^ iNeurons lacking either MUL1 or MARCH5. We describe a possible role for USP30 in translocon quality control in the Discussion.

## Discussion

**Figure 7:**
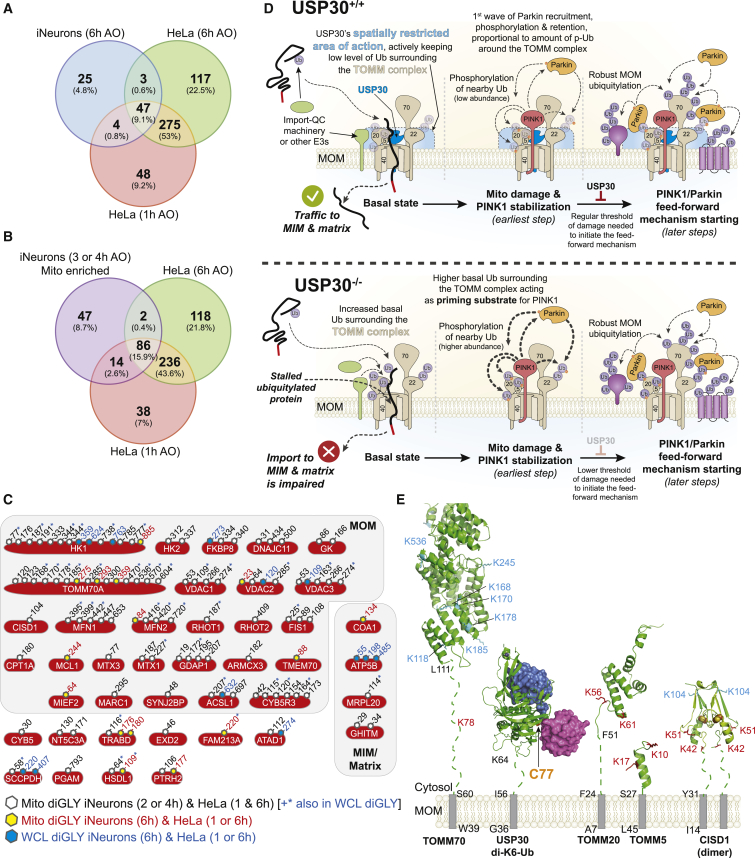
Landscape of Parkin-Dependent Ubiquitylation and USP30-Dependent Deubiquitylation on Mitochondria in iNeurons (A) Venn diagram of overlapping diGLY sites observed in whole-cell lysates (WCLs) (6 h post-depolarization) from iNeurons and sites observed for purified mitochondria for HeLa cells 1 or 6 h post-depolarization. All peptides used were increased by at least 1-fold (log_2_ ratio > 1.0), with p < 0.05. (B) Venn diagram of overlapping diGLY sites observed in purified mitochondria (3 and/or 4 h post-depolarization) from iNeurons and sites observed for purified mitochondria for HeLa cells 1 or 6 h post-depolarization. All peptides used were increased by at least 1-fold (log_2_ ratio > 1.0), with p < 0.05. (C) Diagram showing the sites of ubiquitination in 41 proteins identified as high-confidence Parkin substrates in iNeurons and the corresponding identifications in previously reported data using HeLa cells ectopically expressing Parkin with a parallel TMT-MS^3^ platform (Rose et al., 2016). Residue numbers for diGLY modified Lys residues are shown. Black font and open hexagon, diGLY site found in purified mitochondria from iNeurons (3 and/or 4 h post-depolarization; Table S2) and in purified mitochondria from HeLa cells (1 or 6 h post-depolarization). Sites in black font also noted by the blue asterisk were also found in whole-cell lysates from iNeurons (6 h post-depolarization; Table S1). Red font and yellow hexagon, diGLY site found in purified mitochondria from iNeurons (3 and/or 4 h post-depolarization; Table S2) and in purified mitochondria from HeLa cells depolarized for 1 or 6 h. Blue font and blue hexagon, diGLY site found in whole-cell lysates from iNeurons (6 h post-depolarization; Table S1) and in purified mitochondria from HeLa cells depolarized for 1 or 6 h. (D) Model for USP30-dependent mitochondrial protein deubiquitylation. (E) Structural constraints for USP30-dependent removal of Ub from MOM proteins. Transmembrane segments, gray rectangles. Membrane proximal linkers, dotted lines. Sites of ubiquitylation (red) are those that are reversed by USP30 under depolarization conditions, while those in cyan are largely unchanged in *USP30*^*−/−*^ cells. USP30 catalytic cysteine, yellow. TOMM70, modeled on *S.c.* Tom70, PDB: 2GW1; USP30 in complex with K6-di-Ub, PDB: 5OHP; CISD1 model, PDB: 3EW0; TOMM5 model, PDB: 3PRM; TOMM20 model, PDB: 1OM2. See also Figure S7 and Tables S1 and S3.

Here we report a systematic proteomic analysis of endogenous Parkin action in iNeurons. To define the landscape of Parkin action, we compared Parkin site specificity in iNeurons with our data from HeLa cells with overexpressed Parkin, also using a diGLY/TMT-MS^3^ workflow (Rose et al., 2016). In total, we identified 115 individual diGLY sites in 41 proteins whose abundance increased by 2-fold or more in depolarized iNeurons and that were also found in HeLa cells (Figures 7A–7C). As expected, the largest number of sites (102) were found in common when comparing mitochondrially enriched ubiquitylomes (Figure 7B). The majority of sites (90 sites in 27 proteins) identified were on the cytoplasmic domains of MOM proteins (Figure 7C). In total, 45 individual sites were found in common between the iNeurons whole-cell lysate and enriched mitochondria datasets, the majority being MOM proteins. The pattern of ubiquitylation found with VDACs largely matches the pattern observed previously in iNeurons using targeted proteomics (Ordureau et al., 2018). Taken together, these data provide a set of high confidence Parkin targets, define site specificity at endogenous Parkin levels and suggest the potential for Parkin overexpression to produce ubiquitylation events not present with endogenous Parkin levels.

Early studies of USP30 focused on reversal of Parkin-dependent MOM ubiquitylation in HeLa cells overexpressing Parkin, as well as in *Drosophila*, in which reduction in USP30 function enhanced the activity of Parkin mutants (Bingol et al., 2014, Cunningham et al., 2015, Ordureau et al., 2014). Subsequent studies revealed that USP30 has selectivity for cleavage of K6 linkages and that this activity is inhibited by S65 phosphorylation of the distal Ub in a chain (Gersch et al., 2017, Sato et al., 2017). This, together with the finding that PINK1 preferentially phosphorylates distal Ub moieties within a K6 chain (Gersch et al., 2017), suggests that USP30 will become less effective at removing Ub during depolarization because of increased stoichiometry of Ub phosphorylation (Ordureau et al., 2014). Moreover, analysis of the effect of loss of USP30 on Parkin-independent forms of mitophagy suggests that USP30 may remove pre-existing ubiquitylation events under basal conditions (Marcassa et al., 2018), potentially controlling the Ub threshold required for Parkin activation (Gersch et al., 2017).

In iNeurons, although loss of USP30 promoted Parkin-dependent ubiquitylation of several proteins associated with the translocon, the majority of ubiquitylation events were unaffected. With sub-threshold levels of depolarization, pS65-Ub and mitophagic flux were modestly accelerated, as expected if USP30 sets a threshold for Parkin activation. However, even in cells expressing USP30, Parkin can rapidly overcome this restraint mechanism, presumably as a result of the PINK1-dependent feedforward loop (Figure 7D) (Okatsu et al., 2015, Ordureau et al., 2014, Ordureau et al., 2015). The finding that USP30 action is directed toward the translocon, including TOMM5, TOMM20, TOMM40/L, and one site in TOMM70, is consistent with association of USP30 with the translocon, as shown previously in HeLa cells (Liang et al., 2015) and here in iNeurons (Figure 6A). In addition, the acceleration of pS65-Ub accumulation is also consistent with components of the translocon being important for providing Ub for initial burst of phosphorylation by PINK1, as predicted by the finding that overexpression of a TOMM20^K56A/K61A/K68A^ mutant reduces the rate of mitophagy in HeLa cells (Bingol et al., 2014). However, despite a 2- to 4-fold increase in occupancy of ubiquitylation on TOMM20 under basal conditions (Figure 5D), the stoichiometry of modification was nevertheless at near undetectable levels by immunoblotting (Figures 5B and S7A), suggesting that these sites are not being extensively ubiquitylated in the absence of USP30 under basal conditions. Mitophagic flux iNeuron reporter cells will be useful for testing PINK1 patient mutants in future studies.

In *USP30*^*−/−*^ iNeurons under basal conditions, we unexpectedly observed increased ubiquitylation of proteins normally imported into the matrix or inner membrane via the translocon (Figures S6E, S6F, and S7A). One explanation for increased ubiquitylation is that USP30 functions in a quality control pathway for proteins transiting the translocon (Figure 7D). Removal of ubiquitylation events on such proteins would be expected to be required for their passage through the TOMM40 pore, and in the absence of USP30, import substrates may accumulate in ubiquitylated forms (Figures 7D, S6E, and S6F). We found that CS is mono-ubiquitylated in *USP30*^*−/−*^ but not WT iNeurons under basal conditions; this ubiquitylation was unchanged with depolarization and was not dependent upon either MUL1 or MARCH5, suggesting the involvement of other E3s. Interestingly, loss of MUL1 or MARCH5 also did not affect basal or depolarization-dependent ubiquitylation of TOMM20 or MFN2, nor did it affect the accumulation of pS65-Ub (Figure S7A), indicating that neither E3 significantly controls basal ubiquitylation for priming of PINK1 in iNeurons. We cannot, however, rule out the possibility that MARCH5 and MUL1 function redundantly to promote basal MOM ubiquitylation in this setting or play a modulatory role. Although inhibition of USP30 has also been linked to mitochondrial fission-fusion cycles via regulation of MFNs (Yue et al., 2014), we did not observe changes in MFN1/2 ubiquitylation upon deletion of USP30 in iNeurons (Figures 5B and 5F). Recent work suggests a role for Parkin and PINK1 in import substrate translocation, possibly through the assembly of K11 UB chains (Jacoupy et al., 2019), and further studies are required to understand interplay between Parkin and USP30 in this regard.

Our results suggest a proximity model governing USP30 specificity for removal of Ub from MOM proteins. Given the seven amino acid linker (residues 57–63) connecting the USP30 catalytic domain (residues 64–517) to its transmembrane segment (residues 36–56) together with the orientation and location of its catalytic triad, USP30 action may be limited to ubiquitylation sites that are membrane proximal, with an estimated reach of ∼35 Å from the membrane (Gersch et al., 2017) (Figures 7D and 7E). Although structural data are limited, a working model would suggest that isopeptide substrates substantially further than 35 Å would be challenging for USP30 to reach. We modeled the cytosolic domains of TOMM5, TOMM20, TOMM70, and CISD1 with regions linking folded domains to the transmembrane segment modeled in an extended conformation. With the exception of TOMM70, the folded domains of the substrates were separated from the transmembrane segment by a small number of residues (TOMM20 [residues F24–F51], TOMM5 [residues S26–S27], CISD1 [residues Y31–N41], and Tomm70 [residues S60–L111]) (Figure 7E). Sites that are regulated by USP30 in TOMM5, TOMM20, and CISD1 appear to be compatible with removal, as does K78 in TOMM70, which is also regulated by USP30 (Figures 5D and 5F). However, the many other ubiquitylated sites on TOMM70 that are not regulated by USP30 may be too far away for removal by USP30.

Although numerous studies with Parkin overexpression suggested a dramatic loss in several MOM proteins in a proteasome-dependent manner upon depolarization (Chan et al., 2011, Tanaka et al., 2010), we find that p97-dependent loss of protein abundance in iNeurons is limited to MFN1/2 and RHOT1 but that the reduction in abundance after 6 h is less than 30%. Moreover, ubiquitylation of other Parkin targets and the accumulation of pS65-Ub is unaffected under conditions where MFNs cannot be removed from the MOM via p97 or degraded by the proteasome (Figure 4). These results suggest that the proposed gating mechanism (McLelland et al., 2018) may not be operative in iNeurons with endogenous Parkin levels. Together, this work provides a quantitative landscape for Parkin-dependent mitochondrial ubiquitylation and defines the contributions of USP30 and p97 at endogenous protein levels. This iNeuron system coupled with quantitative proteomics should facilitate biochemical studies aimed at understanding the mechanisms underlying neurodegenerative diseases linked with organelle or protein quality control.

## STAR★Methods

### Key Resources Table

**Table:** 

REAGENT or RESOURCE	SOURCE	IDENTIFIER
**Antibodies**		

Anti-RHOT1	Sigma-Aldrich	Cat# HPA010687; RRID:AB_1079813
Anti-MFN2	Abcam	Cat#ab124773; RRID:AB_10999860
Anti-CISD1	Proteintech	Cat#16006-1-AP; RRID:AB_2080268
Anti-PCNA	Santa-Cruz	Cat#sc-56; RRID:AB_628110
Anti-TOMM20	Santa-Cruz	Cat#sc-11415; RRID:AB_2207533
Anti-TOMM70	Abcam	Cat#ab106193; RRID:AB_10860518
Anti-Parkin	Santa-Cruz	Cat#sc-32282; RRID:AB_628104
Anti-Ubiquitin	Dako	Cat#Z0458; RRID:AB_2315524
Anti-PINK1	Abgent	Cat#AW5456; RRID: N/A
Anti-pS65-Ub	Millipore	Cat#ABS1513; RRID: N/A
Anti-HSP90	Santa-Cruz	Cat#sc-69703; RRID:AB_2121191
Anti-β-Actin	Santa-Cruz	Cat#sc-69879; RRID:AB_1119529
Anti-VDAC1	Abcam	Cat#ab15895; RRID:AB_2214787
Anti-MAP2	Thermo Fisher Scientific	Cat#MA1-19426; RRID:AB_1076853
Anti-USP30	Santa-Cruz	Cat#sc-515235; RRID: N/A
Anti-Keima-Red	MBL international	M182-3; RRID:AB_10794910
Anti-MUL1	Abcam	Cat#ab209263; RRID: N/A
Anti-MARCH5	LifeSpan BioSciences	Cat#LS-C358404; RRID: N/A
Anti-MARCH5	Millipore	Cat#06-1036; RRID:AB_10807027
Anti-Citrate Synthase	Cell Signaling Technology	Cat#14309S; RRID:AB_2665545
Anti-MAP2 (D5G1)	Cell Signaling Technology	Cat# 8707S; RRID: AB_2722660
Anti-β3-Tubulin (TU-20)	Cell Signaling Technology	Cat# 4466S; RRID: N/A
Anti-Parkin (phospho-S65)	The Michael J. Fox Foundation	Cat#MJF-17-42-4; RRID: N/A
Anti-Rabbit IgG (H+L), HRP Conjugate	Promega	Cat# W4011; RRID:AB_430833
Anti-Mouse IgG (H+L), HRP Conjugate	Promega	Cat# W4021; RRID:AB_430834
PTMScan Ubiquitin Remnant Motif (K-ε-GG) (D4A7)	Cell Signaling Technology	Custom order
IRDye 680 RD Goat anti-Mouse IgG H^+^L	LI-COR	926-68070; RRID:AB_10956588

**Bacterial and Virus Strains**		

Rosetta(DE3)pLysS Competent Cells	Novagen	Cat#70956

**Chemicals, Peptides, and Recombinant Proteins**		

Oligomycin A	Sigma-Aldrich	75351
Antimycin A	Sigma-Aldrich	A8674
Digitonin	Gold Biotechnology	D-180
Aprotinin	Roche	10981532001
Leupeptin	Roche	11017101001
AEBSF	Gold Biotechnology	A-540
TCEP	Gold Biotechnology	TCEP2
Doxycycline	Sigma-Aldrich	D9891
Puromycin	Gold Biotechnology	P-600-500
Hygromycin B	Thermo Fisher Scientific	10687010
Hydrogen Peroxide	Sigma-Aldrich	H1009
Formic Acid	Sigma-Aldrich	94318
DAPI	Thermo Fisher Scientific	D1306
CB-5083	Anderson et al., 2015 paper	N/A
NMS-873	Cayman Chemical	17674
Recombinant SpCas9	Zuris et al., 2015; This paper	N/A
Recombinant AsCpf1	Hur et al., 2016; This paper	N/A
Adenosine 5′ triphosphate, disodium, trihydrate (ATP)	Thermo-Fisher Scientific	Cat#10326943
Brain-derived neurotrophic factor (BDNF)	Peprotech	Cat#450-02
Neurotrophin-3	Peprotech	Cat#450-03
Cultrex 3D Culture Matrix Laminin I	R&D Systems	Cat#3446-005-01
Y-27632 Dihydrochloride	Peprotech	Cat#1293823
Trysin	Promega	V511C
Lys-C	Wako Chemicals	129-02541
Rapigest SF Surfactant	Glixx Laboratories	Cat#GLXC-07089
EPPS	Sigma-Aldrich	Cat#E9502
2-Chloroacetamide	Sigma-Aldrich	C0267
Iodoacetamide	Sigma-Aldrich	I1149
PR-619	Selleck Chem	Cat#S7130
Protein A Plus Ultralink resin	Thermo-Fisher Scientific	Cat#53142
LysoTracker Deep Red	Thermo-Fisher Scientific	Cat#L12492
35 mm Dish No. 1.5	MatTek	P35G-1.5-14-C

**Critical Commercial Assays**		

*GeneArt Precision gRNA Synthesis Kit*	Thermo-Fisher	A29377
Pierce High pH Reversed-Phase Peptide Fractionation Kit	Thermo Fisher Scientific	Cat# 84868
High-Select Fe-NTA Phosphopeptide Enrichment Kit	Thermo Fisher Scientific	Cat#A32992
Tandem Mass Tags	Thermo Fisher Scientific	Cat#90406
Quantitative Colorimetric Peptide Assay	Thermo Fisher Scientific	Cat#23275
Bio-Rad Protein Assay Dye Reagent Concentrate	Bio-Rad	Cat##5000006

**Experimental Models: Cell Lines**		

HeLa Flip-In T-REx PARKIN^WT^	Ordureau et al., 2014, Ordureau et al., 2015	N/A
H9 ES cells	WiCell, Madison WI	WA09
H9 ES cells + AAVS-*NGN2*	This paper	N/A
H9 ES *PRKN*^*S65A*^ + AAVS-*NGN2*	Ordureau et al., 2018	N/A
H9 ES *PRKN*^*H302A*^ + AAVS-*NGN2*	This paper	N/A
H9 ES *PINK1*^*−/−*^ + AAVS-*NGN2*	This paper	N/A
H9 ES *USP30*^*−/−*^ + AAVS-*NGN2*	This paper	N/A
H9 ES *USP30*^*−/−*^;*MUL1*^*−/−*^ + AAVS-NGN2	This paper	N/A
H9 ES *USP30*^*−/−*^;*MARCH5*^*−/−*^ + AAVS-NGN2	This paper	N/A

**Oligonucleotides**		

Primers for 5′ AAVS junction PCR	5′-CTCTAACGCTGCCGTCTCTC and 5′-TGGGCTTGTACTCGGTCATC	N/A
Primers for 3′ AAVS junction PCR	5′-CACACAACATACGAGCCGGA and 5′-ACCCCGAAGAGTGAGTTTGC	N/A
Primers for locus PCR	5′-AACCCCAAAGTACCCCGTCT and 5′-CCAGGATCAGTGAAACGCAC	N/A
PARKIN S65A knock-in sgRNA for cutting	5′-GAACAATGCTCTGCTGATCC-3′	N/A
PARKIN S65A knock-in replacement oligonucleotide (bases in caps indicate mutated bases)	5′-tttctggggtcgtcgcctccagttgcattcatttcttga ccttttctccacggtctctgcacaatgtgaac aatgGCctgctgatccaggtcacaattctgttt gggagcaaggtaaaaaaaaaaaaaaaaaa aaaggaaatgtcaaacatg-3′	N/A
USP30 deletion sgRNA	5′- GATATAAAGTCATGAAGAACTGGG-3′	N/A
MUL1 deletion sgRNA	5′-CTGCAAGGGGGTAATTCAG-3′	N/A
MARCH5 deletion sgRNA	5′-GGTCCAGTGGTTTACGTCT-3′	N/A
PARKIN H302A knock-in sgRNA for cutting	5′-TAAAGAGCTCCATCACTTC-3′	N/A
PARKIN H302A knock-in replacement oligonucleotide (bases in caps indicate mutated bases)	5′-gagtgaaagtgacgtttttgtgattaattcttctttc caacagctggctgtcccaactccttgattaaag agctcGCtcacttcaggattctgggagaagag caggtgagtgagcatctcaaaggctgcatcag actgtcatgaaagataga-3′	N/A

**Recombinant DNA**		

pET-NLS-Cas9-6xHis	Zuris et al., 2015; Addgene	62934
pDEST-his-AsCpf1-EC	Hur et al., 2016; Addgene	79007
pAAVS1-TRE3G-EGFP	Addgene	52343
gRNA_AAVS1-T2	Addgene	41818
pAAVS1-TRE3G-NGN2	This study	N/A
hCAS9 plasmid	Addgene	41815
pAC150-PBLHL-4xHS-EF1a – pDEST PiggyBac	Cheng et al., 2013; Addgene	48234
pAC150-PBLHL-4xHS-EF1a – mtx-mKeima^XL^	This study	N/A
pAC150-PBLHL-4xHS-EF1a – mtx-QC(mCherry-GFP)^XL^	This study	N/A
pCMV-hyPBase – hyperactive piggyBac transposase	Yusa et al., 2011	https://www.sanger.ac.uk/form/Sanger_CloneRequests

**Software and Algorithms**		

PyMOL	The PyMOL Molecular Graphics System, v.1.8.6.0, Schrodinger, LLC	https://pymol.org/2/
Prism	GraphPad, v8	https://www.graphpad.com/scientific-software/prism/
In-house mass spectrometry data analysis software	Huttlin et al. Cell. (2010) 143:1174-89.	N/A
SEQUEST	Eng et al., 1994	N/A
Comet	Eng et al., 2013	http://comet-ms.sourceforge.net/
Perseus	Tyanova et al., 2016 13:731-40.	http://maxquant.net/perseus/
FiJi	ImageJ V.2.0.0	https://imagej.net/Fiji
ImageStudioLite	V 5.2.5	https://www.licor.com/bio/image-studio-lite/

**Other**		

Orbitrap Fusion Lumos Mass Spectrometer	ThermoFisher Scientific	Cat#IQLAAEGAAPFADBMBHQ
Easy-nLC 1200	ThermoFisher Scientific	LC140
Aeris 2.6 μm PEPTIDE XB-C18 100 Å 250 × 4.6 mm	Phenomenex	Cat#00G-4505-E0
Yarra 3 μm SEC-2000, LC Column 300 × 4.6 mm	Phenomenex	Cat#00H-4512-E0
HALO Protein C4, 3.4um, 400A	Mac-Mod Analytical	Cat#943410214
Corning Matrigel Matrix, Growth Factor Reduced	Corning	Cat# 354230
Neon Transfection System	Thermo Fisher Scientific	Cat#MPK5000
Maxisorp Plates (96-well)	Sigma-Aldrich	Cat#M9410
Sep-Pak tC18 1cc Vac Cartridge, 50 mg	Waters	Cat#WAT054960
SOLA HRP SPE Cartridge, 10 mg	Thermo Fisher Scientific	Cat#60109-001
Empore SPE Disks C18	3M Bioanalytical Technologies	Cat# 2215
Empore SPE Disks C8	3M Bioanalytical Technologies	Cat#2214
Bio-Rad Protein Assay Dye Reagent Concentrate	Bio-Rad	Cat#5000006
Odyssey CLx Imager	LI-COR bioscience	N/A

### Lead Contact and Materials Availability

Further information and requests for resources and reagents should be directed to and will be fulfilled by the Lead Contact, J. Wade Harper (wade_harper@hms.harvard.edu)

### Experimental Model and Subject Details

#### Gene-Editing and differentiation

Human ES cells (H9, WiCell Institute) were cultured in E8 medium (Chen et al., 2011) on Matrigel-coated tissue culture plates with daily medium change. Cells were passaged every 4-5 days with 0.5 mM EDTA in 1 × DPBS (Thermo Fisher Scientific). SpCas9 and AsCas12a/AsCpf1 expression plasmids pET-NLS-Cas9-6xHis (Addgene plasmid # 62934) and modified pDEST-his-AsCpf1-EC, generated by deleting the MBP sequence from plasmid pDEST-hisMBP-AsCpf1-EC (Addgene plasmid # 79007), were transformed into Rosetta(DE3)pLysS Competent Cells (Novagen), respectively. SpCas9 and AsCas12a/AsCpf1 proteins were purified as described elsewhere (Hur et al., 2016, Zuris et al., 2015). The sgRNAs were generated using GeneArt Precision gRNA Synthesis Kit (Thermo Fisher Scientific) according to the manufacturer’s instruction and purified using RNeasy Mini Kit (QIAGEN). The sgRNA target sequences were: *MUL1*^*−/−*^, CTGCAAGGGGGTAATTCAG; *MARCH5*^*−/−*^, GGTCCAGTGGTTTACGTCT; PRKN^H302A^, TAAAGAGCTCCATCACTTC.

H9 cells harboring homozygous Parkin^S65A^ alleles were described previously (Ordureau et al., 2018). To generate *MUL1*^*−/−*^ and *MARCH5*^*−/−*^ H9 ES cells, 0.6 μg sgRNA was incubated with 3 μg SpCas9 protein for 10 minutes at room temperature and electroporated into 2x10^5^ H9 cells using Neon transfection system (Thermo Fisher Scientific). To generate *USP30*^*−/−*^ H9 ES cells, 80 pmol Alt-R® CRISPR-Cpf1 crRNA targeting GATATAAAGTCATGAAGAACTGGG (IDT) was incubated with 63 pmol AsCas12a/AsCpf1 protein for 10 minutes at room temperature and electroporated into 2x10^5^ H9 cells, along with 39 pmol Alt-R® Cpf1 Electroporation Enhancer (IDT). To create H9 cells harboring a homozygous H302A mutation in *PRKN*, 0.6 μg sgRNA was incubated with 3 μg SpCas9 protein for 10 minutes at room temperature and electroporated into 2x10^5^ H9 cells along with a ssDNA oligo (gagtgaaagtgacgtttttgtgattaattcttctttccaacagctggctgtcccaactccttgattaaagagctcGCtcacttcaggattctgggagaagagcaggtgagtgagcatctcaaaggctgcatcagactgtcatgaaagataga). Mutants were identified by Illumina MiSeq and further confirmed by western blot for *MUL1*^*−/−*^, *MARCH5*^*−/−*^ and *USP30*^*−/−*^, and Sanger sequencing for *PRKN*^*H302A*^. For introduction of TRE3G-*NGN2* into the AAVS1 site, a donor plasmid pAAVS1-TRE3G-*NGN2* was generated by replacing the EGFP sequence with N-terminal flag-tagged human *NGN2* cDNA sequence in plasmid pAAVS1-TRE3G-EGFP (Addgene plasmid # 52343). Five micrograms of pAAVS1-TRE3G-*NGN2*, 2.5 μg hCas9 (Addgene plasmid # 41815), and 2.5 μg gRNA_AAVS1-T2 (Addgene plasmid # 41818) were electroporated into 1x10^6^ H9 cells. The cells were treated with 0.25 μg/ml Puromycin for 7 days and surviving colonies were expanded and subjected to genotyping. The primers for 5′ junction PCR were: 5′-CTCTAACGCTGCCGTCTCTC and 5′-TGGGCTTGTACTCGGTCATC. The primers for 3′ junction PCR were 5′-CACACAACATACGAGCCGGA and 5′-ACCCCGAAGAGTGAGTTTGC. The primers for locus PCR were 5′-AACCCCAAAGTACCCCGTCT and 5′-CCAGGATCAGTGAAACGCAC.

H9 ES cells harboring the mitochondrial matrix mKeima or mCherry-GFP flux reporter were generated by electroporation of 1x10^6^ cells with 2.5 μg of pAC150-PiggyBac-matrix-mKeima^XL^ or pAC150-PiggyBac-matrix-mCherry-eGFP^XL^ along with 2.5 μg of pCMV-HypBAC-PiggyBac-Helper. The cells were selected and maintained in E8 medium supplemented with 50 μg/ml Hygromycin and Hygromycin was kept in the medium during differentiation to iNeurons.

For human ES cell conversion to iNeurons, cells were expanded and plated at 2x10^4^/cm^2^ on Matrigel-coated tissue plates in DMEM/F12 supplemented with 1x N2, 1x NEAA (Thermo Fisher Scientific), human Brain-derived neurotrophic factor (BDNF, 10 ng/ml, PeproTech), human Neurotrophin-3 (NT-3, 10 ng/l, PeproTech), mouse laminin (0.2 μg/ml, Cultrex), Y-27632 (10 μM, PeproTech) and Doxycycline (2 μg/ml, Alfa Aesar) on Day 0. On Day 1, Y-27632 was withdrawn. On Day 2, medium was replaced with Neurobasal medium supplemented with 1x B27 and 1x Glutamax (Thermo Fisher Scientific) containing BDNF, NT-3 and 1 μg/ml Doxycycline. Starting on Day 4, half of the medium was replaced every other day thereafter. On Day 7, the cells were treated with Accutase (Thermo Fisher Scientific) and plated at 3-4x10^4^/cm^2^ on Matrigel-coated tissue plates. Doxycycline was withdrawn on Day 10. The extent of differentiation was monitored using α-β3-Tubulin (TUBB3), and α-MAP2 immunostaining 12-14 days post-differentiation. The following differentiation efficiencies were determined by counting triplicate cultures: WT: 99.0 ± 0.07 (n > 93); S65A-Parkin: 92.0 ± 1.7 (n > 25); H302A-Parkin 99.1 ± 1.3 (n > 22); USP30−/−: 97.5 ± 2.2 (n > 25).

#### Cloning and generation of stable mitophagic flux reporter hESCs lines

The sequence encoding the tandem dual repeat (for enhanced specificity) of the mitochondrial-targeting sequence of COX VIII (Rudolf et al., 2004), was used to direct mKeima (Katayama et al., 2011) or QC (mCherry-eGFP) (Allen et al., 2013) reporters to the mitochondrial matrix. The mtx-mKeima^XL^ probe (1032 bp) consist of the matrix-targeting tandem sequence, followed by a 3xFlag tag sequence, a V5-tag sequence and ending with mKeima sequence (Katayama et al., 2011) (Figure 6C). The mtx-QC^XL^ probe (1803 bp) consist of the matrix-targeting tandem sequence, followed by a mCherry sequence, a 3xFlag tag sequence, a V5-tag sequence and ending with eGFP sequence (Figure 6C). All the individual elements, with the exception of mKeima, were human codon-optimized to minimize repeats and redundancy at the codon level and facilitate subsequent cloning. The probes of interest were cloned into pDonor223 vector and transferred to piggyBac pDEST vector (pAC150-PBLHL-4xHS-EF1a – addgene #48234).

### Method Details

#### Cell culture, immunoblotting, and mitochondrial protein isolation

HeLa Flip-In T-REx cells (generously provided by Brian Raught, Ontario Cancer Institute) engineered to inducibly express a single copy of Parkin^WT^, the catalytically defective Parkin^C431S^ mutant, or a non-phosphorylatable Parkin^S65A^ mutant were created as described previously (Ordureau et al., 2014, Ordureau et al., 2015). The indicated cells (HeLa or iNeurons) were either left untreated or depolarized with a mixture of Antimycin A (10 μM) and Oligomycin A (5 μM) for the indicated time period. At the indicated times, cells were washed twice with ice cold PBS and lysed in lysis buffer (50 mM Tris/HCl pH 7.5, 1 mM EDTA, 1 mM EGTA, 50 mM NaF, 5 mM sodium pyrophosphate, 10 mM sodium 2-glycerol 1-phosphate, 1% (v/v) NP-40, 1 μg/ml aprotinin, 1 μg/ml leupeptin, 1 mM benzamidine, 1 mM AEBSF, 10 μM PR-619, 50 mM chloroacetamide and 1x PhosSTOP phosphatase inhibitor Cocktail (Roche)), to produce whole cell extracts. In some experiments, cells were incubated with one of two p97 inhibitors [NMS-873 (5 μM) or CB-5083 (5 μM)] (Anderson et al., 2015, Magnaghi et al., 2013).

Crude mitochondria was purified after two washes in ice cold PBS by scraping cells in PBS containing 100 mM chloroacetamide (3 mL per 15 cm dish). Cells were then collected and centrifuged at 450 × g for 5 minutes at 4°C. Cell pellet was resuspended in mitochondrial isolation buffer (MIB) (50 mM Tris/HCl, pH 7.5, 70 mM Sucrose, 210 mM Sorbitol, 1 mM EDTA, 1 mM EGTA, 50 mM NaF, 5 mM sodium pyrophosphate, 10 mM sodium 2-glycerophosphate, 1 mM AEBSF, 10 μM PR-619, 1 mM benzamidine, 1 μg/ml leupeptin and aprotinin) plus 100 mM chloroacetamide and centrifuged at 1400 × g for 5 minutes at 4°C. The cell pellet was re-suspended in MIB buffer plus 100 mM chloroacetamide and sonicated twice for 10 s at lowest settings. Samples were spun 10 min at 1400 × g to remove unbroken cells/debris. The supernatant was collected, this correspond to the “total protein” fraction, and transferred into a round-bottom tube prior to centrifugation for 10 min at 10000 × g at 4°C. Supernatant which correspond to cytosolic fraction and crude ER fraction was removed and the pellet corresponding to the crude mitochondria fraction was resuspended in MIB buffer, prior to centrifugation for 10 min at 10000 × g. The mitochondrial pellet was washed two more times and the pellet was then lysed in lysis buffer.

For intact mass analysis, mitochondria were purified with 25 mM iodoacetamide in place of chloroacetamide and the last two washes were performed without iodoacetamide.

Whole cell extracts or mitochondrial extracts were sonicated and clarified by centrifugation (16000 × g for 10 min at 4°C) followed by filtration through a 0.45 μM filter and protein concentrations determined by the Bradford assay. Samples were denatured by the addition of LDS sample buffer supplemented with 100 mM DTT, followed by boiling at 75°C for 5 minutes. Cell extracts (25 μg or 50 μg for p-S65-Ub immunoblotting) or mitochondrial extracts were separated using 4%–12% NuPAGE Bis-Tris gel (Thermo Fisher Scientific), using MOPS of MES SDS running buffer (Thermo Fisher Scientific) and proteins were electro-transferred to PVDF membranes (0.45 μm, Millipore). The membrane was then blocked with 5% non-fat milk, incubated with the indicated primary antibodies, washed three times with TBST (total 30 min), and further incubated with the relevant secondary HRP-conjugated antibody. After several washes with TBST for 30 min, signal was detected using enhanced chemiluminescence and X-ray film. For mito-flux quantification, fluorescent IRDye 680RD Goat anti-Mouse IgG H^+^L secondary antibody (1:20000) for 1 hour was used. After several washes with TBST for 30 min, near infrared signal was detected using OdysseyCLx imager and quantified using ImageStudioLite (LI-COR).

#### Immunoprecipitation of TOMM20 and USP30 protein

Crude mitochondria was purified after two washes in ice cold PBS, and 2 washes in hypotonic buffer (20 mM HEPES (pH 7.8), 5 mM KCl, 1.5 mM MgCl2, 0.5 mM TCEP, 1 μg/ml aprotinin, 1 μg/ml leupeptin, 1 mM AEBSF) by scraping cells in hypotonic buffer. Cells collected and left on ice for 15 min prior to dounce homogenization. “2.5xMSH” buffer (525 mM mannitol, 175 mM sucrose, 20 mM HEPES (pH 7.8), 5 mM EDTA) was mix with cell homogenate to reach a final 1xMSH concentration. Lysate were then centrifuged at 700 × g for 10 minutes at 4°C to remove cell debris and nuclei. Supernatant was transferred to new tube and spun at 8500 × g for 10 minutes at 4°C to pellet mitochondria. Pellet was resuspended in 1xMSH buffer (210 mM mannitol, 70 mM sucrose, 20 mM HEPES, 2 mM EDTA) and spun at 8500 × g for 10 minutes at 4°C to pellet mitochondria. Mitochondria were resuspended in 100 μl of 1xMSH buffer supplemented with 1% digitonin and were homogenized by ten passes through a 21-gauge (1.25 inches long) needle and incubated at 4°C with gentle agitation for 5 min. The homogenate was sedimented by centrifugation at 16000 × g for 5 min and the supernatant was transferred to a new tube and protein concentrations determined by the Bradford assay. The TOMM20, USP30 or IgG control antibodies were coupled to Protein A Plus Ultralink resin (1:1 μL slurry/ μg antibody) (Thermo Fisher Scientific) overnight at 4°C prior to its dimethyl pimelimidate chemical cross-linking reaction. The cross-linked antibody beads were washed twice in PBS and twice in 1xMSH buffer supplemented with 0.1% digitonin. 50 μg of crude mitoprep were mixed with 4 ul of antibody-beads and adjusted to a final volume of 500 μl using 1xMSH buffer supplemented with 0.1% digitonin. Mixture was incubated for 4 hours at 4°C with gentle end-over-end rotation. After centrifugation at 215 × g for 2 min, immune complexes were washed three times with 1xMSH buffer supplemented with 0.1% digitonin and 200 mM NaCl and once with 10 mM Tris/HCl (pH 8.0). The immune complexes were transfer to Spin-X centrifuge tube filters (Corning Costar) and the bound proteins were eluted with 1x NuPAGE LDS sample buffer in the absence of any thiol. The beads were centrifuged for 1 min at 6000 × g and the flowthrough collected, supplemented with 100 mM DTT, followed by boiling at 75°C for 5 minutes and subjected to SDS-PAGE.

#### Immunoprecipitation of diGLY-Containing Peptides

diGLY capture was performed largely as described (Rose et al., 2016) . The diGly monoclonal antibody (Cell Signaling Technology; D4A7 clone) (32 μg antibody/1 mg peptide) was coupled to Protein A Plus Ultralink resin (1:1 μL slurry/ μg antibody) (Thermo Fisher Scientific) overnight at 4°C prior to its chemical cross-linking reaction. Dried peptides (indicated amount in corresponding figures) were resuspended in 1.5 mL of ice-cold IAP buffer [50 mM MOPS (pH 7.2), 10 mM sodium phosphate and 50 mM NaCl] and centrifuged at maximum speed for 5 min at 4°C to remove any insoluble material. Supernatants (pH ∼7.2) were incubated with the antibody beads for 2 hr at 4°C with gentle end-over-end rotation. After centrifugation at 215 × g for 2 min, beads were washed three more times with ice-cold IAP buffer and twice with ice-cold PBS. The diGLY peptides were eluted twice with 0.15% TFA, desalted using homemade StageTips and dried via vacuum centrifugation, prior to TMT labeling.

#### Proteomics – General Sample Preparation

Protein extracts were subjected to disulfide bond reduction with 5 mM TCEP (room temperature, 10 min) and alkylation with 25 mM chloroacetamide (room temperature, 20 min). Methanol–chloroform precipitation was performed prior to protease digestion. In brief, four parts of neat methanol were added to each sample and vortexed, one part chloroform was then added to the sample and vortexed, and finally three parts water was added to the sample and vortexed. The sample was centrifuged at 6 000 rpm for 2 min at room temperature and subsequently washed twice with 100% methanol. Samples were resuspended in 100 mM EPPS pH8.5 containing 0.1% RapiGest and digested at 37°C for 4h with LysC protease at a 200:1 protein-to-protease ratio. Trypsin was then added at a 100:1 protein-to-protease ratio and the reaction was incubated for a further 6 h at 37°C. Samples were acidified with 1% Formic Acid for 15 min and subjected to C18 solid-phase extraction (SPE) (Sep-Pak, Waters). The Pierce Quantitative Colorimetric Peptide Assay (cat.no. 23275) was used to quantify the digest and to accurately aliquot the desired amount of peptides (indicated in corresponding figures) per sample needed for downstream application.

#### Proteomics – Total proteomics analysis using TMT

Tandem mass tag labeling of each sample (100 μg peptide input) was performed by adding 10 μL of the 20 ng/μL stock of TMT reagent along with acetonitrile to achieve a final acetonitrile concentration of approximately 30% (v/v). Following incubation at room temperature for 1 h, the reaction was quenched with hydroxylamine to a final concentration of 0.5% (v/v) for 15 min. The TMT-labeled samples were pooled together at a 1:1 ratio. The sample was vacuum centrifuged to near dryness, and subjected to C18 solid-phase extraction (SPE) (50 mg, Sep-Pak, Waters).

Dried TMT-labeled sample was resuspended in 100 μL of 10 mM NH_4_HCO_3_ pH 8.0 and fractionated using BPRP HPLC (Wang et al., 2011). Briefly, samples were offline fractionated over a 90 min run, into 96 fractions by high pH reverse-phase HPLC (Agilent LC1260) through an aeris peptide xb-c18 column (Phenomenex; 250 mm x 3.6 mm) with mobile phase A containing 5% acetonitrile and 10 mM NH_4_HCO_3_ in LC-MS grade H_2_O, and mobile phase B containing 90% acetonitrile and 10 mM NH_4_HCO_3_ in LC-MS grade H_2_O (both pH 8.0). The 96 resulting fractions were then pooled in a non-continuous manner into 24 fractions (as outlined in Figure S5 of Paulo et al. 2016a) and 12 fractions (even numbers) were used for subsequent mass spectrometry analysis. Fractions were vacuum centrifuged to near dryness. Each consolidated fraction was desalted via StageTip, dried again via vacuum centrifugation, and reconstituted in 5% acetonitrile, 1% formic acid for LC-MS/MS processing.

Mass spectrometry data were collected using an Orbitrap Fusion Lumos mass spectrometer (Thermo Fisher Scientific, San Jose, CA) coupled to a Proxeon EASY-nLC1200 liquid chromatography (LC) pump (Thermo Fisher Scientific). Peptides were separated on a 100 μm inner diameter microcapillary column packed in house with ∼35 cm of Accucore150 resin (2.6 μm, 150 Å, ThermoFisher Scientific, San Jose, CA) with a gradient consisting of 5%–21% (0-125 min), 21%–28% (125-140min) (ACN, 0.1% FA) over a total 150 min run at ∼500 nL/min. For analysis, we loaded 1/10 of each fraction onto the column. Each analysis used the Multi-Notch MS^3^-based TMT method (McAlister et al., 2014), to reduce ion interference compared to MS^2^ quantification (Paulo et al., 2016b). The scan sequence began with an MS^1^ spectrum (Orbitrap analysis; resolution 120,000 at 200 Th; mass range 400−1400 m/z; automatic gain control (AGC) target 5 × 10^5^; maximum injection time 50 ms). Precursors for MS^2^ analysis were selected using a Top10 method. MS^2^ analysis consisted of collision-induced dissociation (quadrupole ion trap analysis; Turbo scan rate; AGC 2.0 × 10^4^; isolation window 0.7 Th; normalized collision energy (NCE) 35; maximum injection time 90 ms). Monoisotopic peak assignment was used and previously interrogated precursors were excluded using a dynamic window (150 s ± 7 ppm) and dependent scans were performed on a single charge state per precursor. Following acquisition of each MS^2^ spectrum, a synchronous-precursor-selection (SPS) MS^3^ scan was collected on the top 10 most intense ions in the MS^2^ spectrum (McAlister et al., 2014). MS^3^ precursors were fragmented by high energy collision-induced dissociation (HCD) and analyzed using the Orbitrap (NCE 65; AGC 3 × 10^5^; maximum injection time 150 ms, resolution was 50,000 at 200 Th).

#### Proteomics – Phospho proteomics analysis using TMT

Phospho peptides were enriched using Pierce Fe-NTA phosphopeptide enrichment kit (A32992) and following the provided protocol. In brief, dried peptides, unlabeled (Figure 1) or TMT-labeled (Figures 4 and 5) were enriched for phosphopeptides, while the unbound peptides and washes were saved for proteome analysis (Figures 4 and 5) or diGLY enrichment (Figure 1) after C18 solid-phase extraction (SPE). The enriched phosphopeptides were dried down and desalted via StageTip prior to MS analysis (Figure 5), or fractionated (Figures 1 and 4) according to manufacturer’s instructions using High pH reversed-phase peptide fractionation kit (Thermo Fisher Scientific) for a final 6 fractions and subjected to C18 StageTip desalting prior to MS analysis.

Mass spectrometry data were collected using an Orbitrap Fusion Lumos mass spectrometer (Thermo Fisher Scientific, San Jose, CA) coupled to a Proxeon EASY-nLC1200 liquid chromatography (LC) pump (Thermo Fisher Scientific). Peptides were separated on a 100 μm inner diameter microcapillary column packed in house with ∼35 cm of Accucore150 resin (2.6 μm, 150 Å, ThermoFisher Scientific, San Jose, CA) with a gradient consisting of 5%–16% (0-78 min), 16%–22% (78-98min), 22%–28% (98-110 min) (ACN, 0.1% FA) over a total 120 min at ∼500 nL/min. For analysis, we loaded 1/2 of each fraction onto the column. Each analysis used the Multi-Notch MS^3^-based TMT method (McAlister et al., 2014). The scan sequence began with an MS^1^ spectrum (Orbitrap analysis; resolution 120,000 at 200 Th; mass range 400−1400 m/z; automatic gain control (AGC) target 1 × 10^6^; maximum injection time 50 ms). Precursors for MS^2^ analysis were selected using a Top10 method. MS^2^ analysis consisted of collision-induced dissociation (quadrupole ion trap analysis; Turbo scan rate; AGC 2.0 × 10^4^; isolation window 0.7 Th; normalized collision energy (NCE) 35; maximum injection time 150 ms) with MultiStage Activation (MSA) for neutral loss of 97.9763. Monoisotopic peak assignment was used and previously interrogated precursors were excluded using a dynamic window (150 s ± 7 ppm). Following acquisition of each MS^2^ spectrum, a synchronous-precursor-selection (SPS) MS^3^ scan was collected on the top 10 most intense ions in the MS^2^ spectrum (McAlister et al., 2014). MS^3^ precursors were fragmented by high energy collision-induced dissociation (HCD) and analyzed using the Orbitrap (NCE 65; AGC 1.5 × 10^5^; maximum injection time 250 ms, resolution was 50,000 at 200 Th).

#### Proteomics – diGLY proteomics analysis using TMT

TMT-labeled diGLY peptides were fractionated according to manufacturer’s instructions using High pH reversed-phase peptide fractionation kit (Thermo Fisher Scientific) for a final 6 fractions and subjected to C18 StageTip desalting prior to MS analysis.

Mass spectrometry data were collected using an Orbitrap Fusion Lumos mass spectrometer (Thermo Fisher Scientific, San Jose, CA) coupled to a Proxeon EASY-nLC1200 liquid chromatography (LC) pump (Thermo Fisher Scientific). Peptides were separated on a 100 μm inner diameter microcapillary column packed in house with ∼35 cm of Accucore150 resin (2.6 μm, 150 Å, ThermoFisher Scientific, San Jose, CA) with a gradient consisting of 3%–26% (0-130 min), 26%–32% (130-140min) (ACN, 0.1% FA) over a total 150 min run at ∼500 nL/min. For analysis, we loaded 1/2 of each fraction onto the column. Each analysis used the Multi-Notch MS^3^-based TMT method (McAlister et al., 2014). The scan sequence began with an MS^1^ spectrum (Orbitrap analysis; resolution 120,000 at 200 Th; mass range 400−1250 m/z; automatic gain control (AGC) target 1 × 10^6^; maximum injection time 100 ms). Precursors for MS^2^ analysis were selected using a Top 4 s method. MS^2^ analysis consisted of collision-induced dissociation (quadrupole Orbitrap analysis; AGC 1 × 10^5^; isolation window 0.7 Th; normalized collision energy (NCE) 35; maximum injection time 300 ms resolution was 7,500 at 200 Th). Monoisotopic peak assignment was used and previously interrogated precursors were excluded using a dynamic window (120 s ± 7 ppm). As described previously, only precursors with a charge state between 3 and 6 were selected for downstream analysis (Rose et al., 2016). Following acquisition of each MS^2^ spectrum, a synchronous-precursor-selection (SPS) MS^3^ scan was collected on the top 10 most intense ions in the MS^2^ spectrum (McAlister et al., 2014). MS^3^ precursors were fragmented by high energy collision-induced dissociation (HCD) and analyzed using the Orbitrap (NCE 65; AGC 2 × 10^5^; maximum injection time 500 ms, resolution was 50,000 at 200 Th).

#### Proteomics – Data analysis

Mass spectra were processed using a Sequest-based or Comet-based (2014.02 rev. 2) in-house software pipeline (Eng et al., 2013, Huttlin et al., 2010). Spectra were converted to mzXML using a modified version of ReAdW.exe. Database searching included all entries from the Human Reference Proteome (2017-05) UniProt database, as well as an in-house curated list of contaminants. This database was concatenated with one composed of all protein sequences in the reversed order. Searches were performed using a 20 ppm precursor ion tolerance for total protein level analysis. The product ion tolerance was set to 0.9 Da (0.03 Da for diGLY searches). These wide mass tolerance windows were chosen to maximize sensitivity in conjunction with Sequest searches and linear discriminant analysis (Beausoleil et al., 2006, Huttlin et al., 2010). TMT tags on lysine residues and peptide N termini (+229.163 Da) and carbamidomethylation of cysteine residues (+57.021 Da) were set as static modifications, while oxidation of methionine residues (+15.995 Da) was set as a variable modification. For phosphorylation dataset search, phosphorylation (+79.966 Da) on Serine or Threonine and deamidation (+0.984 Da) on Asparagine or Glutamine were set as additional variable modifications. For diGLY dataset search, GlyGly modification (+114.0429 Da) was also set as a variable modification. Peptide-spectrum matches (PSMs) were adjusted to a 1% false discovery rate (FDR) (Elias and Gygi, 2007). PSM filtering was performed using a linear discriminant analysis, as described previously (Huttlin et al., 2010), while considering the following parameters: XCorr (or Comet Log Expect), ΔCn (or Diff Seq. Delta Log Expect), missed cleavages, peptide length, charge state, and precursor mass accuracy. For TMT-based reporter ion quantitation, we extracted the summed signal-to-noise (S:N) ratio for each TMT channel and found the closest matching centroid to the expected mass of the TMT reporter ion (integration tolerance of 0.003 Da). For protein-level comparisons, PSMs were identified, quantified, and collapsed to a 1% peptide false discovery rate (FDR) and then collapsed further to a final protein-level FDR of 1%. Moreover, protein assembly was guided by principles of parsimony to produce the smallest set of proteins necessary to account for all observed peptides. Phosphorylation or ubiquitylation site localization was determined using the AScore algorithm (Beausoleil et al., 2006). AScore is a probability-based approach for high-throughput protein phosphorylation site localization. Specifically, a threshold of 13 corresponded to 95% confidence in site localization. Proteins and phosphorylated or ubiquitylated peptides were quantified by summing reporter ion counts across all matching PSMs using in-house software, as described previously (Huttlin et al., 2010). PSMs with poor quality, MS^3^ spectra with isolation specificity less than 0.7, or with TMT reporter summed signal-to-noise ratio that were less than 150, or had no MS_3_ spectra were excluded from quantification (McAlister et al., 2012).

Protein or peptide quantification values were exported for further analysis in Microsoft Excel, GraphPad Prism and Perseus (Tyanova et al., 2016). Each reporter ion channel was summed across all quantified proteins and normalized assuming equal protein loading of all samples. Supplemental data Tables list all quantified proteins as well as associated TMT reporter ratio to control channels used for quantitative analysis.

Annotations for bona fide organellar protein markers were assembled using the proteins which had scored with confidence “very high” or “high” from the HeLa dataset previously published Itzhak D.N. (Itzhak et al., 2016). The following database containing mitochondrial protein were used: MitoCarta 2.0 (Calvo et al., 2016) and the Mitochondrial Outer Membrane (MOM) dataset from (Hung et al., 2017).

#### Proteomics – LbPro^∗^ sample preparation

Mitochondrial pellets were resuspended in 50 mM Tris/HCl pH 8.0 buffer containing 0.1% NP40 and protease inhibitors as 1x PhosSTOP phosphatase inhibitor Cocktail (Roche). Mitochondrial extracts were sonicated and clarified by filtration through a 0.45 μM filter and protein concentrations determined by the Bradford assay. Extracts (250 μg for HeLa cells and 75 μg for iNeurons) were diluted to a volume of 100-150 μL containing a final concentration of 25 mM Tris/HCl (pH 8.0), 25 mM NaCl, 10 mM DTT and 10 μM Lbpro^∗^ (Swatek et al., 2019). Extract were digested overnight at 37°C with discontinuous agitation (1200 RPM). Next morning, 2 μL aliquot were separated by SDS/PAGE, transferred to PVDF membranes and probed for ubiquitin to ensure complete digestion and disappearance of Ub signal above the size of mono-ubiquitin (8.5 kDa). Samples were clarified by centrifugation (16000xg for 5 min at room temperature) followed by filtration through a 0.45 μM filter and transferred to HPLC sample glass vial.

Samples were offline fractionated over a 40 min run, by size exclusion using an HPLC (Agilent LC1260) through a Yarra 3 μm SEC-2000 column (Phenomenex; 300 mm x 4.6 mm) with mobile phase containing 20% acetonitrile and 20 mM HEPES, 150 mM NaCl in LC-MS grade H_2_O (pH 7.2). The fractions containing cleaved ubiquitin were then pooled and used for subsequent mass spectrometry analysis. Fractions were acidified with formic acid and subjected to C8 StageTip desalting, dried again via vacuum centrifugation, and reconstituted in 5% acetonitrile, 0.1% formic acid for LC-MS/MS processing.

Mass spectrometry data were collected using an Orbitrap Fusion Lumos mass spectrometer (Thermo Fisher Scientific, San Jose, CA) coupled to a Proxeon EASY-nLC1200 liquid chromatography (LC) pump (Thermo Fisher Scientific). Peptides were separated on a 100 μm inner diameter microcapillary column packed in house with ∼35 cm of HALO Protein C4 resin (3.4 um, 400 Å) (Mac-Mod Analytical) with a gradient consisting of 10%–16% (0-2 min), 16%–18% (2-5min), 18%–30% (5-20min) (ACN, 0.1% FA) over a total 30 min run at ∼800 nL/min. For analysis, we loaded 1/2 of each fraction onto the column. The instrument was operated in standard pressure mode, default charge state set to 10 and Advanced Peak Determination (APD) was used. The scan sequence began with an MS^1^ spectrum (Orbitrap analysis with quadrupole isolation; resolution 60,000 at 200 Th; mass range 625−925 m/z; automatic gain control (AGC) target 3 × 10^5^; maximum injection time 50 ms, 3 micro scans per scan and data collected in profile mode). Monoisotopic peak assignment was not used and previously interrogated precursors were excluded using a dynamic window (15 s ± 15 ppm if occurred twice in 10 s). Precursors for MS^2^ analysis (quality control purpose only and not used for downstream analysis/quantification) were selected from an inclusion list containing the various proteoforms of interest (m/z value for z = 12 of the isotope form containing 5 C13) using a Top 2.5 s method. MS^2^ analysis consisted of collision-induced dissociation (quadrupole Orbitrap analysis; AGC 5.0 × 10^5^; isolation window 1 Th; normalized collision energy (NCE) 35; maximum injection time 54 ms, 3 micro scans per scan, resolution was 15,000 at 200 Th, data collected in profile mode).

#### Proteomics – Intact mass data analysis

Mass spectra were processed using Xcalibur Qual Browser (v4.2 Thermo Fisher Scientific) and the Xtract algorithm (v3.0 Thermo Fisher Scientific) for deconvolution and deisotoping of spectras (mass mode MH+, mono isotopic masses extracted, 189-2000 mass range, signal to noise 3, fit factor 80%, Remainder 25%, averagine no sulfur, and max charge 17). The extracted deconvoluted spectra were then analyzed further using Xcalibur and the peak area of the extracted ion chromatogram (XIC) was determined for each proteoforms of interest (automatic processing, smoothing Gaussian 7 points, 500 mmu mass tolerance, mass precision 4 decimals). Proteoform quantification (XIC peak area) values were exported for further analysis in Microsoft Excel and GraphPad Prism.

#### Proteomics – Heavy reference peptides and characterization

Heavy reference peptides (Table S4) for parallel reaction monitoring containing a single ^13^C/^15^N-labeled amino acid were synthesized, purified by reversed-phase chromatography and quantified by amino acid analysis by Cell Signaling Technologies. Heavy reference peptides from working stocks (in 5% FA / 5% ACN) were diluted into the digested sample (in 1% FA) to be analyzed to an optimal final concentration predetermined for individual peptides. LC retention times, precursor ion to be targeted, isolation window, AGC target, fill time were determined for each peptide (Table S4). A mixture of all AQUA peptides was used to determine the best ACN gradient to minimize peptide overlap during elution in a 90-min gradient. Samples (1 μg of digested purified mitochondria) and heavy reference peptides were oxidized with 0.1% hydrogen peroxide for 30 min, subjected to C18 StageTip desalting and hydrogen peroxide removal, and resuspended in 1% FA / 2.5% ACN. Samples were analyzed sequentially by LC-MS on a Orbitrap Fusion Lumos instrument coupled to an Easy-nLC 1200 (Thermo Fisher Scientific) ultra-high-pressure liquid chromatography (UHPLC) pump. Peptides were separated on a 100 μm inner diameter microcapillary column packed in house with ∼35 cm of Accucore150 resin (2.6 μm, 150 Å, ThermoFisher Scientific, San Jose, CA). The column was equilibrated with buffer A (3% ACN + 0.125% FA). Peptides were loaded onto the column at 100% buffer A. Separation and elution from the column was achieved using a 90-min 3%−23% gradient of buffer B (100% ACN + 0.125% FA) at ∼575 nL/min. The scan sequence began with FTMS^1^ spectra (resolution of 120,000; mass range 325-900 m/z; automatic gain control (AGC) target 1x10^6^, max injection time of 100 ms). The second scan sequence consisted of a targeted-MS^2^ (tMS^2^) method were MS^2^ precursors of interested as defined in the “Mass List Table” (Table S4) were isolated using the quadrupole and analyzed in the Orbitrap (FTMS^2^) with an isolation window, resolution, AGC target and a max injection time defined in the “Mass List Table” (Table S4). MS^2^ precursors were fragmented by HCD at a normalized collision energy (NCE) of 30%. LC-MS data analysis was performed using Skyline software (MacLean et al., 2010) with manual validation of precursors and fragments. The results exported to Excel and GraphPad Prism for further analysis and plotting.

#### Live-cell confocal microscopy for mitophagic flux analysis

Cells were plated onto 33 mm-glass bottom dishes (No. 1.5, 14 mm glass diameter, MatTek) on day 7 of the differentiation. On day 12, the cells were treated with Antimycin A (0.5 μM) and Oligomycin (0.5 μM) for 4, 6, 8, 16, and 24 h, then stained with 25 nM LysoTracker Deep Red (Thermo Fisher Scientific) for 30 minutes prior to the imaging analysis. The media was replaced to pre-warmed fresh media (37°C), and the cells were imaged using a Yokogawa CSU-X1 spinning disk confocal with Spectral Applied Research Aurora Borealis modification on a Nikon Ti motorized microscope equipped with a Nikon Plan Apo 60 × /1.40 N.A objective lens. The cells were maintained in 37°C condition using Okolab cage incubator through-out the imaging acquisition. Images for eGFP, mCherry, and LysoTracker Deep Red fluorescence were collected sequentially using 100 mW 491 nm, 100 mW 561 nm, and 101 mW 642 nm solid state lasers attenuated and controlled with an AOTF (Spectral Applied Research LMM-5), and emission collected with 525/50, 620/60, and 700/75 nm filters (Chroma Technologies), respectively. Confocal images were acquired with the Hamamatsu Flash4.0 V3 sCMOS camera and MetaMorph software. Consistent laser intensity and exposure time were applied to all the samples, and brightness and contrast were adjusted equally by applying the same minimum and maximum display values in FiJi software.

Image Quantitation: For each condition, a total of 10 image sections taken with a 60x objective lens (containing 1-2 soma per section) were analyzed using Fiji software. All the sections were included for the analysis except the cells that showed less than 30% of the GFP-mCherry expression levels compared to the average fluorescent intensity (to remove low level expressing cells). Step 1) An image taken with a 561 ex/620 em set were subtracted by the corresponding 491 ex/525 em set, which resulted in a new image showing mCherry signal only pixels. Step 2) Following the background subtraction, a binary image was generated for ‘mask’ that covers mCherry only area. The binary segment was added to ROI manager. Step3) The mean fluorescent intensity for the images from step1 were obtained applying a mask obtained from step2. ‘Red only signal’ per soma was calculated by applying the following equation (Area x percent area x 0.01 x mean intensity). Each value was normalized by the average value obtained from the untreated condition.

#### Immunocytochemical analysis

The iNeurons were fixed in 4% paraformaldehyde in PBS for 20 min at room temperature and then blocked and permeabilized with 0.25% Triton X-100 and 1% BSA in PBS at room temperature for 1hr. Alternatively, the iNeurons were fixed and permeabilized in cold methanol at −20°C for 20 min and blocked in 2% BSA for 1hr. Anti-MAP2 antibody (Cell Signaling) and anti-β3 tubulin (TUBB3) antibody (Cell Signaling) were diluted at 1:200 in PBS with 0.05% Triton X-100 and 0.1% BSA and applied overnight at 4**°**C. Secondary antibodies (Thermo Fisher Scientific were diluted at 1:1000 in PBS with 0.05% Triton X-100 and 0.1% BSA) and applied for 1 hr at room temperature. Vectashield mounting medium for fluorescence with DAPI (Vector Laboratories) was used for nuclear counterstain. TUBB3^+^ cells in 3 fields were counted for each sample.

#### Structural modeling

Models for proteins were generated in SWISS-MODEL (https://swissmodel.expasy.org/interactive) using the following templates: TOMM70 (*S.c.* Tom70, 2GW1); TOMM5 (3PRM); TOMM20 (1OM2). Structures were rendered in Pymol (https://www.pymol.org/2/).

### Quantification and Statistical Analysis

#### Statistics

All statistical data were calculated using GraphPad Prism 7 or Perseus. Comparisons of data were performed by Welch’s t test corrected for multiple comparison by permutation-based FDR and detailed parameters used are indicated in every figures and tables when applicable.

#### Reproducibility

Unless stated otherwise all quantitative experiments were performed in triplicate and average with SEM reported.

### Data and Code Availability

All data are available by request.

## Supplemental Information

Document S1. Figures S1–S7

Table S1. Total Proteome, Total Phospho-Proteome, and Total Ubiquitylome Data for Parkin^WT^ and Parkin^S65A^ iNeurons with or without Depolarization, Related to Figures 1 and 3

Table S2. Proteome Abundance, Phospho-Proteome, and Ubiquitylome Data for Whole-Cell Extracts from WT iNeurons with or without Depolarization in the Presence or Absence of the Two p97 Inhibitors (CB-5083 or NMS-873), Related to Figure 4

Table S3. Proteome Abundance, Phospho-Proteome, and Ubiquitylome Data for Mitochondria from USP30^+/+^ and USP30^−/−^ iNeurons with or without Depolarization, Related to Figures 5 and 6

Table S4. Light and Heavy Reference Peptide Sequences and Parameters, Related to Figure 6

Document S2. Article plus Supplemental Information
