# Supplementary material for: Global Landscape and Dynamics of Parkin and USP30-Dependent Ubiquitylomes in iNeurons during Mitophagic Signaling
Source: Mol Cell. 2020 Mar 5;77(5):1124–1142.e10. doi: 10.1016/j.molcel.2019.11.013 (PMC7098486; doi:10.1016/j.molcel.2019.11.013)
Supplement: Document S1. Figures S1–S7 [file mmc1.pdf]

Molecular Cell, Volume *77*

Supplemental Information

Global Landscape and Dynamics of Parkin and USP30-Dependent Ubiquitylomes in iNeurons during Mitophagic Signaling

Alban Ordureau, Joao A. Paulo, Jiuchun Zhang, Heeseon An, Kirby N. Swatek, Joe R. Cannon, Qiaoqiao Wan, David Komander, and J. Wade Harper

TITLES OF SUPPLEMENTAL MATERIALS

Supplemental **Figure S1-7**. Relevant to Figures 1-7.

Figure S1. Global proteomic and phospho-proteomic analysis of iNeurons in response to mitochondrial depolarization and Parkin activation. Relevant to Figure 1.

Figure S2. Quantitative analysis of pS65-Ub proteoforms on the MOM in iNeurons using $Ub^{\Delta GG}$ amputation and intact mass spectrometry. Relevant to Figure 2.

Figure S3. Quantitative discovery of the Parkin-dependent ubiquitylome in iNeurons during mitophagic signaling. Relevant to Figure 3.

Figure S4. Quantitative analysis of p97-dependent mitochondrial proteome remodeling and PINK1-Parkin pathway activity in iNeurons. Relevant to Figure 4.

Figure S5. Selectivity of USP30-dependent buffering of MOM ubiquitylation by Parkin in iNeurons. Relevant to Figure 5.

Figure S6. Analysis of the effect of USP30 deficiency on mitochondria in response to depolarization. Relevant to Figure 6.

Figure S7. Mitochondria resident E3 ligases MUL1 and MARCH5 do not modify $USP30^{-/-}$ phenotype and detailed landscape of Parkin-dependent ubiquitylation across cell lines. Relevant to Figure 7.

Figure S1

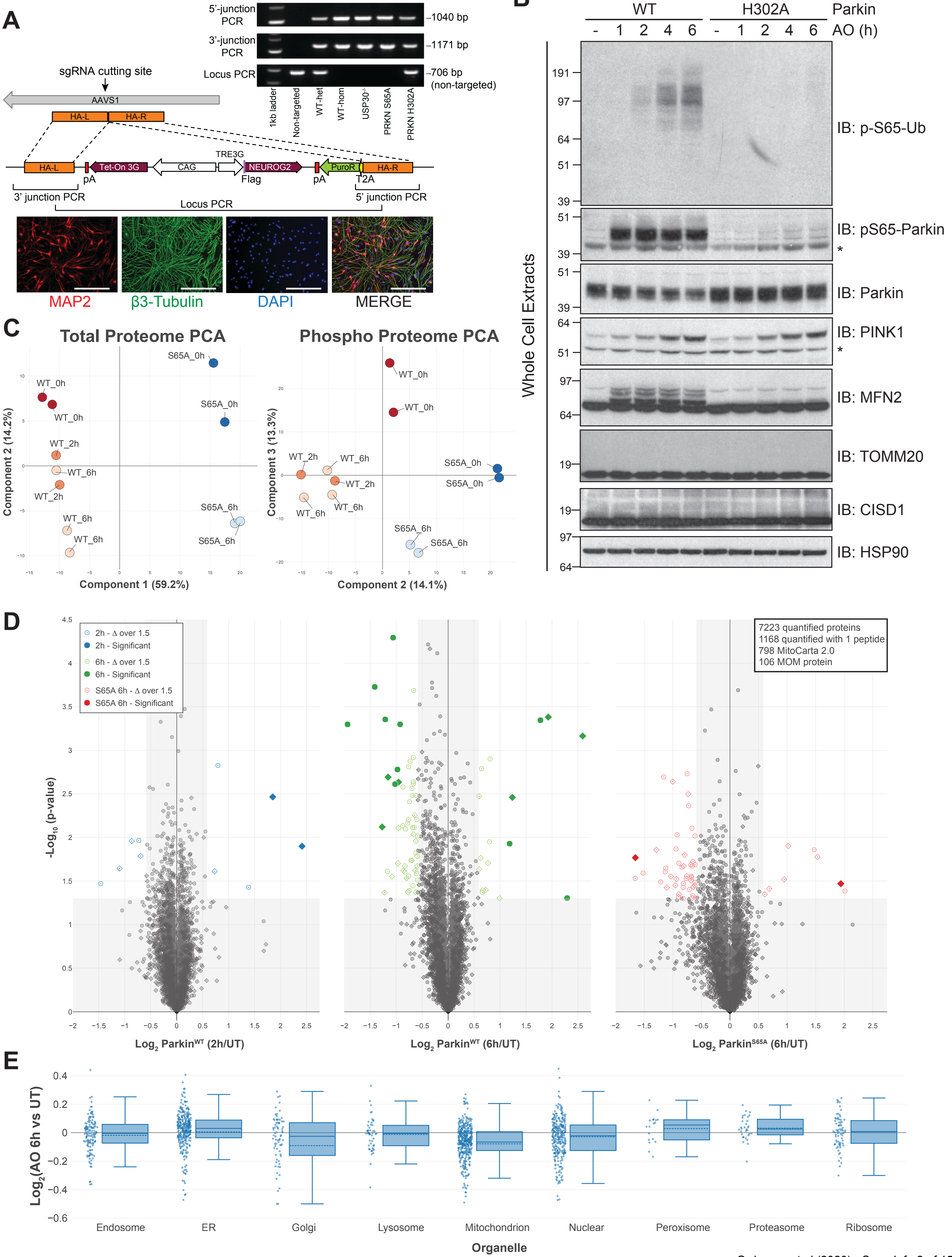
A
5'-junction PCR
3'-junction PCR
Locus PCR
–1040 bp
–1171 bp
–706 bp (non-targeted)
1kb ladder
Non-targeted
WT-het
WT-hom
USP30-/-
PRKN S65A
PRKN H302A
sgRNA cutting site
AAVS1
HA-L
HA-R
Tet-On 3G
CAG
TRE3G
NEUROG2
PuroR
pA
Flag
T2A
3' junction PCR
5' junction PCR
Locus PCR
MAP2
β3-Tubulin
DAPI
MERGE
B
WT
H302A
Parkin
AO (h)
- 1 2 4 6 - 1 2 4 6
Whole Cell Extracts
IB: p-S65-Ub
IB: pS65-Parkin
IB: Parkin
IB: PINK1
IB: MFN2
IB: TOMM20
IB: CISD1
IB: HSP90
C
Total Proteome PCA
Phospho Proteome PCA
Component 1 (59.2%)
Component 2 (14.2%)
Component 2 (14.1%)
Component 3 (13.3%)
WT_0h
WT_2h
WT_6h
S65A_0h
S65A_6h
D
2h - Δ over 1.5
2h - Significant
6h - Δ over 1.5
6h - Significant
S65A 6h - Δ over 1.5
S65A 6h - Significant
7223 quantified proteins
1168 quantified with 1 peptide
798 MitoCarta 2.0
106 MOM protein
-Log10 (p-value)
Log2 ParkinWT (2h/UT)
Log2 ParkinWT (6h/UT)
Log2 ParkinS65A (6h/UT)
E
Log2(AO 6h vs UT)
Endosome
ER
Golgi
Lysosome
Mitochondrion
Nuclear
Peroxisome
Proteasome
Ribosome
Organelle

FIGURE S1. Global proteomic and phospho-proteomic analysis of iNeurons in response to mitochondrial depolarization and Parkin activation. Related to Figure 1.

(A) Creation and validation of iNeurons generation. Scheme of the strategy used to target NGN2 to the AAVS1 locus and resulting PCR products for the various cell line used. Parental H9 hESCs gene edited to express NGN2 under the TRE3G promoter from the AAVS1 locus were induced to express NGN2 for 12 days and the extent of differentiation determined by co-immunostaining for MAP2, β3-Tubulin and DAPI (scale bar = 200 microns).

(B) iNeurons expressing WT or H302A-Parkin were subjected to depolarization and total cell extracts subjected to immunoblotting with the indicated antibodies. *, non-specific band.

(C) Principal component analysis of proteomics data obtained for the 11-plex experiment described in **Figure 1A**.

(D) Volcano plots for total proteomes of WT or S65A-Parkin iNeurons depolarized for 2 or 6h compared with untreated cells. Protein quantified with more than one peptide are represented by a circle, while quantification based on a single peptide are represented by a square. Proteins with Log_2-Ratio < -0.585 or > 0.585 (p-value <0.05) are indicated as colored empty dotted circles/squares, and filled colored circles/squares indicate statistically significant hits (Welch's t-test (S0=0.585), corrected for multiple comparison by permutation-based FDR (5%)).

(E) Plot of Log_2(6h AO/Untreated) for proteins localized in the indicated organelles or protein complexes. Proteins present in each organelle/protein complex were derived from (Itzhak et al., 2016).

Figure S2

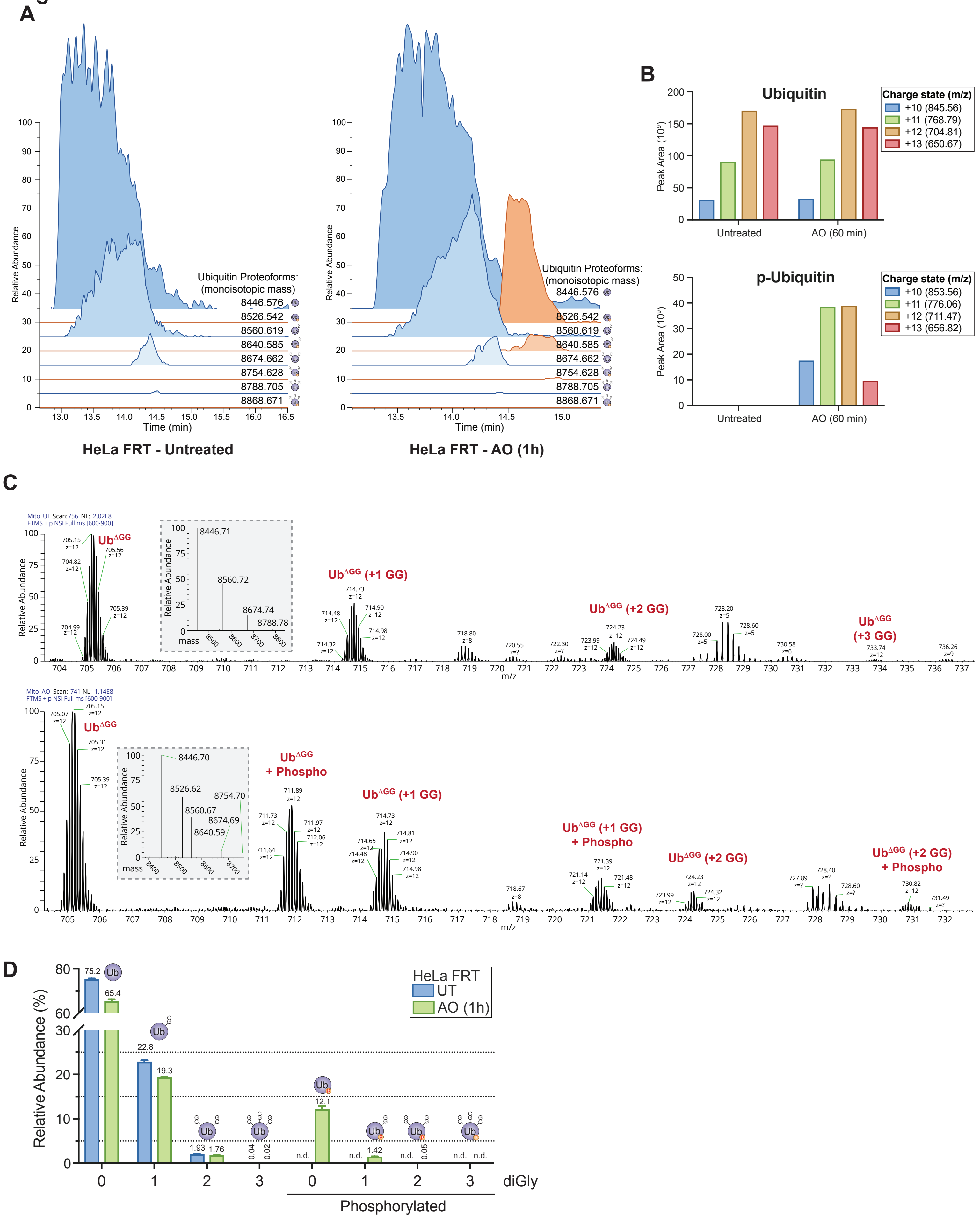
A
Relative Abundance
Ubiquitin Proteoforms:
(monoisotopic mass)
8446.576
8526.542
8560.619
8640.585
8674.662
8754.628
8788.705
8868.671
Time (min)
HeLa FRT - Untreated
HeLa FRT - AO (1h)
B
Ubiquitin
Peak Area (10⁹)
Charge state (m/z)
+10 (845.56)
+11 (768.79)
+12 (704.81)
+13 (650.67)
Untreated
AO (60 min)
p-Ubiquitin
Charge state (m/z)
+10 (853.56)
+11 (776.06)
+12 (711.47)
+13 (656.82)
C
Mito_UT Scan:756 NL: 2.02E8
FTMS + p NSI Full ms [600-900]
Relative Abundance
UbΔGG
UbΔGG (+1 GG)
UbΔGG (+2 GG)
UbΔGG (+3 GG)
8446.71
8560.72
8674.74
8788.78
mass
m/z
Mito_AO Scan: 741 NL: 1.14E8
FTMS + p NSI Full ms [600-900]
UbΔGG
UbΔGG + Phospho
UbΔGG (+1 GG)
UbΔGG (+1 GG) + Phospho
UbΔGG (+2 GG)
UbΔGG (+2 GG) + Phospho
8446.70
8526.62
8754.70
8560.67
8674.69
8640.59
D
Relative Abundance (%)
HeLa FRT
UT
AO (1h)
75.2
65.4
22.8
19.3
1.93
1.76
0.04
0.02
n.d.
12.1
1.42
0.05
diGly
Phosphorylated

FIGURE S2. Quantitative analysis of pS65-Ub proteoforms on the MOM in iNeurons using $Ub^{\Delta GG}$ amputation and intact mass spectrometry. Related to Figure 2.

(A) Deconvoluted extracted ion chromatograms for $Ub^{\Delta GG}$ proteoforms from HeLa Flip-In Parkin cell derived mitochondria with or without depolarization (1h) with AO. Distinct $Ub^{\Delta GG}$ branched diGLY proteoforms in untreated cells largely co-migrate on reversed-phase (C4) LC while phospho-$Ub^{\Delta GG}$ proteoforms elute at a distinct position.

(B) Relative abundance of charge state distributions are different for $Ub^{\Delta GG}$ and pS65-$Ub^{\Delta GG}$, and therefore integration across all charge states is required for quantification.

(C) Examples of mass spectrometry raw spectra showing detection of individual $Ub^{\Delta GG}$ proteoforms in untreated (top) or in response to depolarization (bottom) of HeLa cells expressing $Parkin^{WT}$. Shown are spectra of the isotopic cluster for the z=12 precursors. Deconvoluted spectra and mass of the various proteoforms are indicated in grey boxes.

(D) Relative abundance of $Ub^{\Delta GG}$ proteoforms using the workflow described in **FIGURE 2B**, but applied to HeLa cells. Error bars represent SEM, n=3. n.d., not determined.

Figure S3

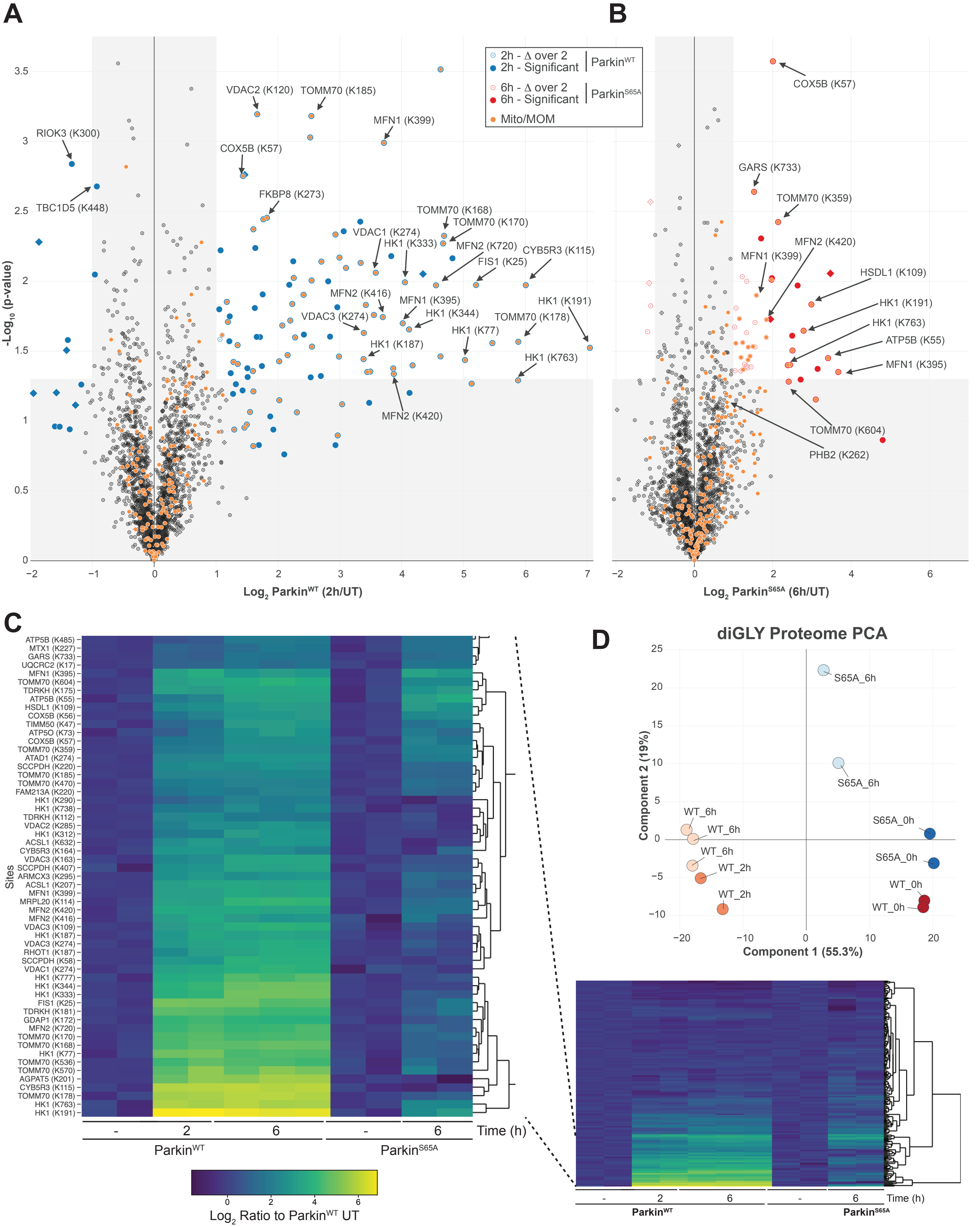
A
B
C
D
2h - Δ over 2
2h - Significant
ParkinWT
6h - Δ over 2
6h - Significant
ParkinS65A
Mito/MOM
-Log10 (p-value)
Log2 ParkinWT (2h/UT)
Log2 ParkinS65A (6h/UT)
RIOK3 (K300)
TBC1D5 (K448)
VDAC2 (K120)
TOMM70 (K185)
MFN1 (K399)
COX5B (K57)
FKBP8 (K273)
TOMM70 (K168)
TOMM70 (K170)
VDAC1 (K274)
HK1 (K333)
MFN2 (K720)
CYB5R3 (K115)
FIS1 (K25)
MFN2 (K416)
MFN1 (K395)
HK1 (K191)
VDAC3 (K274)
HK1 (K344)
TOMM70 (K178)
HK1 (K77)
HK1 (K187)
HK1 (K763)
MFN2 (K420)
GARS (K733)
TOMM70 (K359)
HSDL1 (K109)
ATP5B (K55)
TOMM70 (K604)
PHB2 (K262)
Sites
Time (h)
ParkinWT
ParkinS65A
Log2 Ratio to ParkinWT UT
diGLY Proteome PCA
Component 1 (55.3%)
Component 2 (19%)
S65A_6h
WT_6h
WT_2h
S65A_0h
WT_0h

FIGURE S3. Quantitative discovery of the Parkin-dependent ubiquitylome in iNeurons during mitophagic signaling. Related to Figure 3.

(A,B) Volcano plot of diGLY-containing peptides identified by TMT-MS^3 from whole-cell lysates derived from (A) WT iNeurons with or without depolarization for 2h, or (B) $Parkin^{S65A}$ iNeurons with or without depolarization for 6h. The abundance of diGLY-peptides was normalized against the total protein abundance when available (circle) or not (square). Filled colored circles/squares indicate statistically significant hits (Welch's t-test (S0=1), corrected for multiple comparison by permutation-based FDR (1%)). diGLY-modified residue is indicated in bracket after the protein name. diGLY-peptide of proteins associated with mitochondria (MitoCarta 2.0 (Calvo et al., 2016) or mitochondrial outer membrane identified by proximity biotinylation (Hung et al., 2017)) are overlaid with an orange circle.

(C) Heat map of mitochondrial-diGLY peptides across the WT and S65A-Parkin cells without or with depolarization for 2 or 6h. Only diGLY-peptides of proteins associated with mitochondria (MitoCarta 2.0 (Calvo et al., 2016) or mitochondrial outer membrane identified by proximity biotinylation (Hung et al., 2017)) are represented.

(D) Principal component analysis of diGLY data across the WT and S65A-Parkin cells without or with depolarization for 2 or 6h.

Figure S4

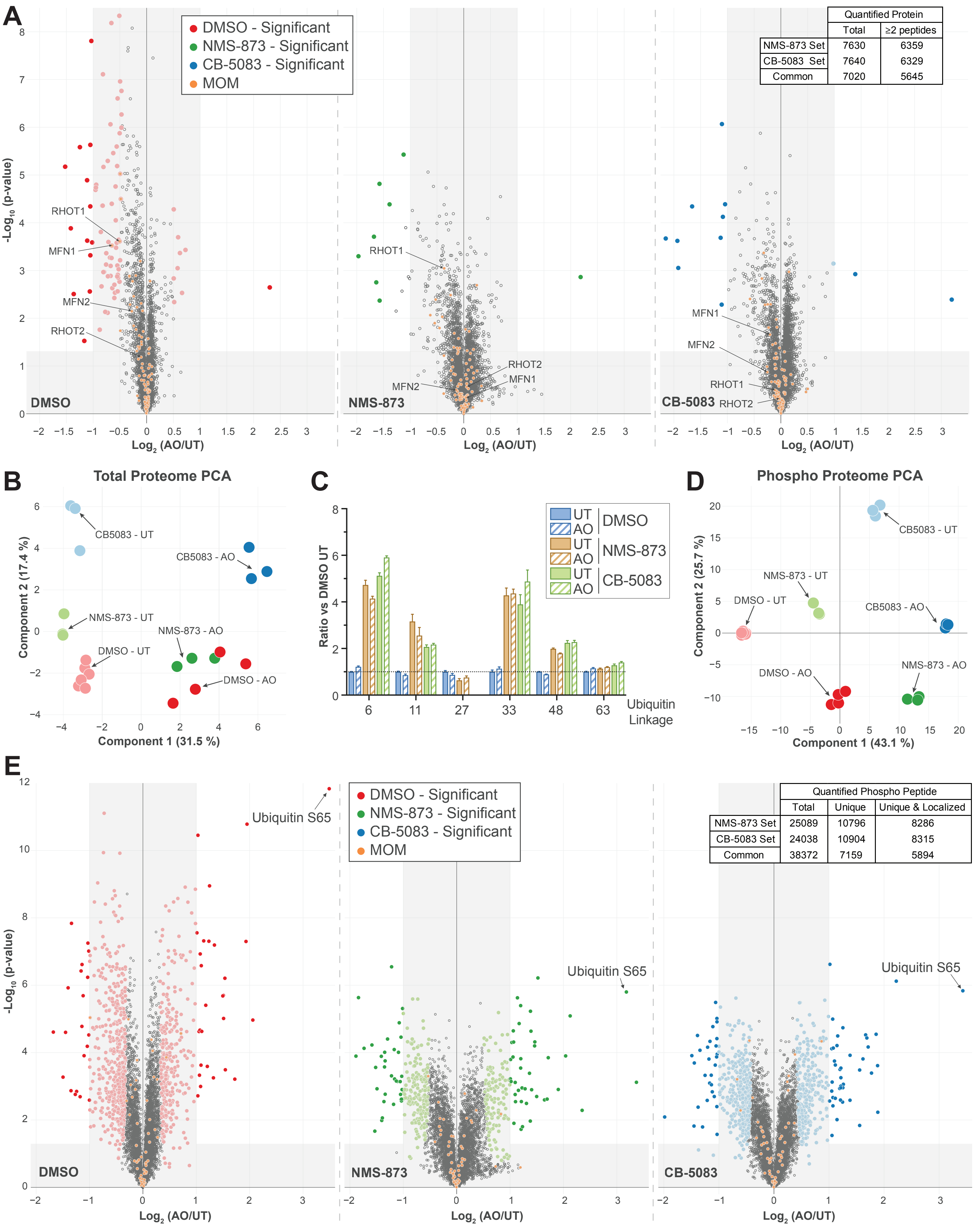

	Quantified Protein	
	Total	≥2 peptides
NMS-873 Set	7630	6359
CB-5083 Set	7640	6329
Common	7020	5645

	Quantified Phospho Peptide		
	Total	Unique	Unique & Localized
NMS-873 Set	25089	10796	8286
CB-5083 Set	24038	10904	8315
Common	38372	7159	5894

FIGURE S4. Quantitative analysis of p97-dependent mitochondrial proteome remodeling and PINK1-Parkin pathway activity in iNeurons. Related to Figure 4.

(A) Volcano plots for total proteomes of iNeurons either left untreated or depolarized for 6h in the presence or absence of the p97 inhibitors NMS-873 or CB-5083. Only proteins quantified in both p97 inhibitor dataset and also quantified with more than one peptide are represented (circle). Filled colored circles/squares indicate statistically significant hits (Welch's t-test (S0=1), corrected for multiple comparison by permutation-based FDR (1%)). Proteins associated with mitochondrial outer membrane identified by proximity biotinylation (Hung et al., 2017)) are overlaid with an orange circle.

(B) PCA analysis of whole proteome data from panel A.

(C) Ub chain linkage types were determined as ratio versus untreated for iNeurons either left untreated or depolarized for 6h in the presence of absence of the p97 inhibitors NMS-873 or CB-5083. Error bars represent SEM, n=3.

(D) PCA analysis of phospho-proteome data from panel E.

(E) Volcano plots for phospho-proteomes of iNeurons either left untreated or depolarized for 6h in the presence of absence of the p97 inhibitors NMS-873 or CB-5083. Only peptides quantified in both p97 inhibitor datasets are represented (circle). Filled colored circles indicate statistically significant hits (Welch's t-test (S0=1), corrected for multiple comparison by permutation-based FDR (1%)). Phospho-peptide of proteins associated with mitochondrial outer membrane identified by proximity biotinylation (Hung et al., 2017) are overlaid with an orange circle.

Figure S5

A

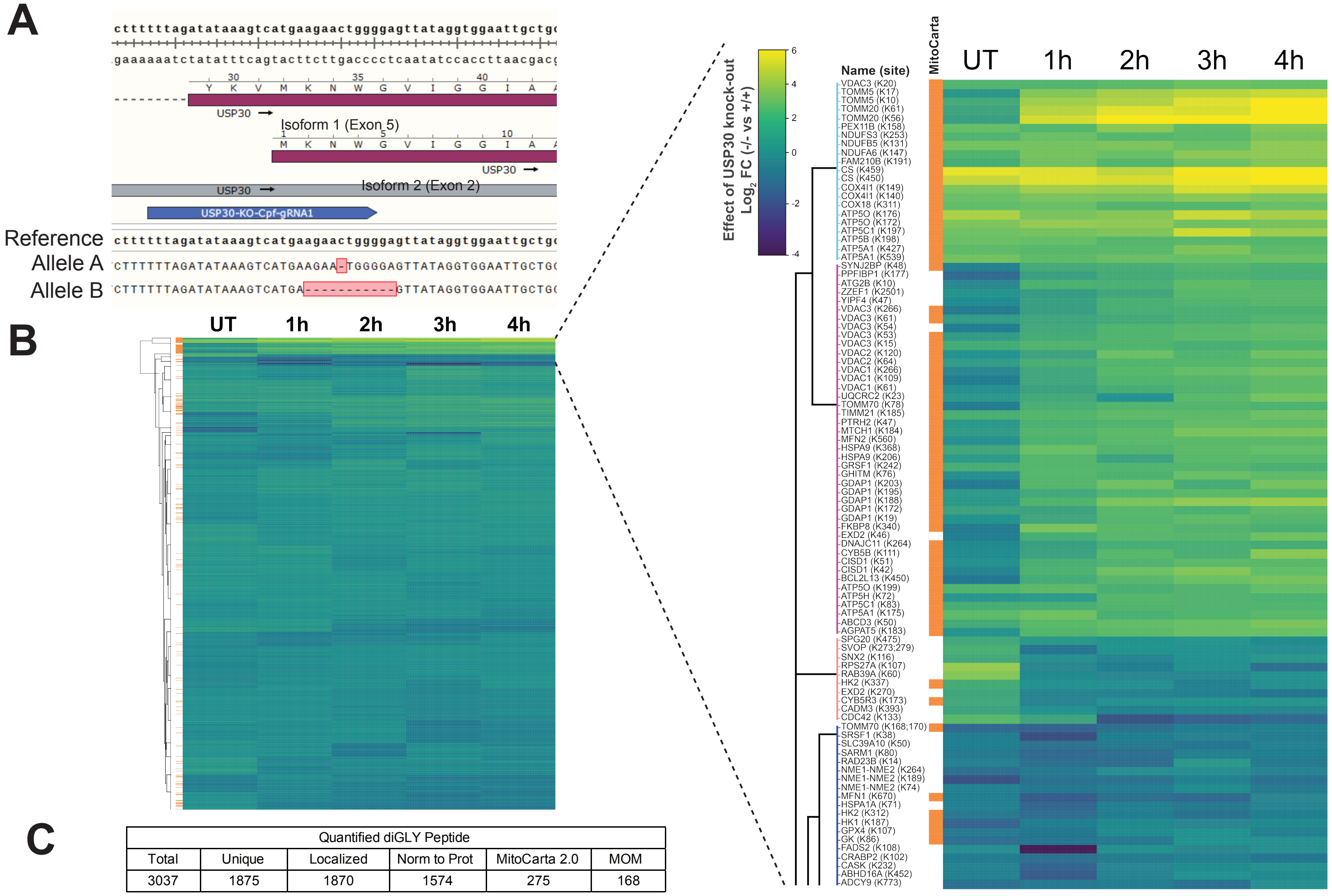

C

Quantified diGLY Peptide					
Total	Unique	Localized	Norm to Prot	MitoCarta 2.0	MOM
3037	1875	1870	1574	275	168

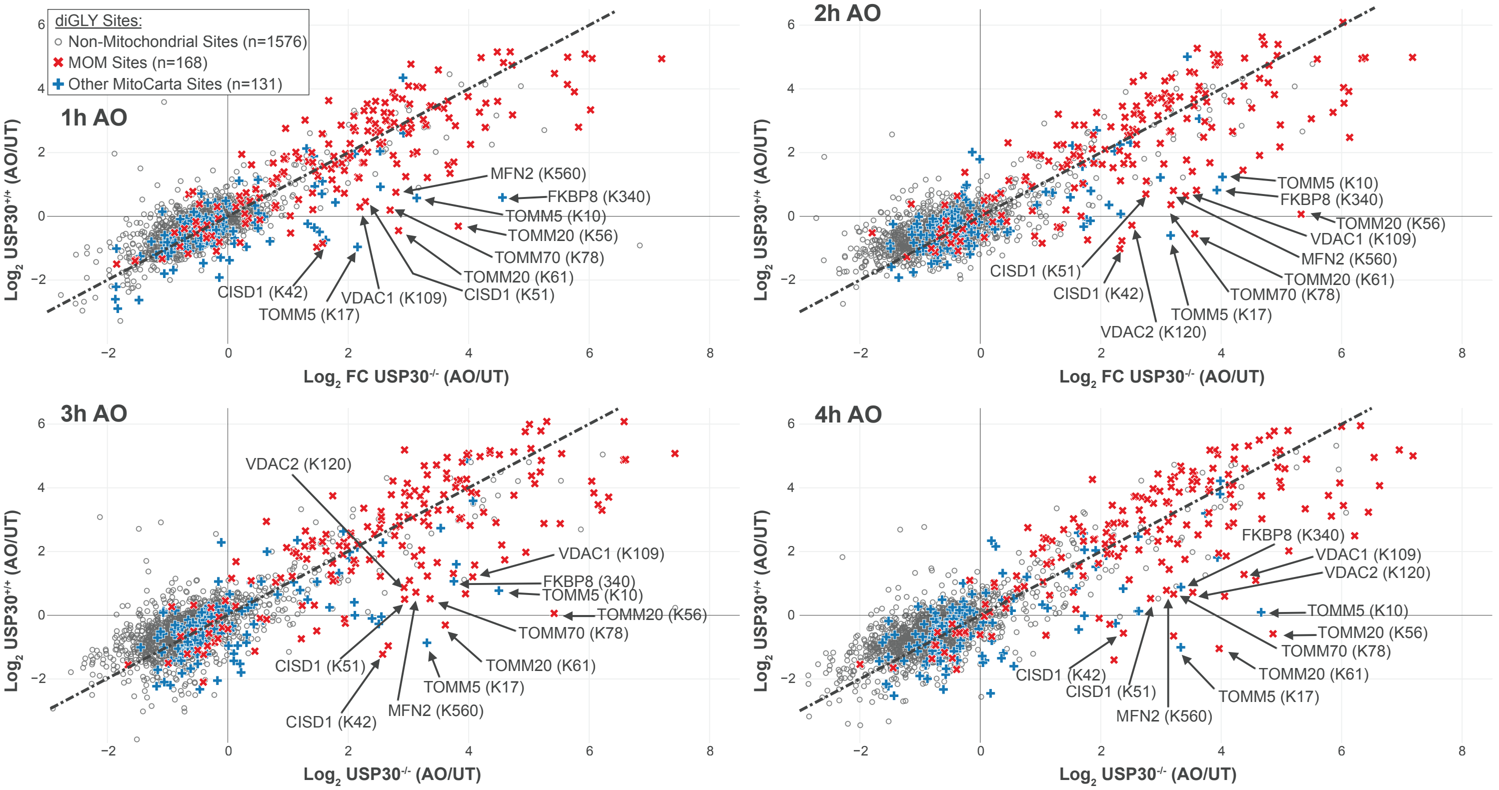

FIGURE S5. Selectivity of USP30-dependent buffering of MOM ubiquitylation by Parkin in iNeurons. Related to Figure 5.

(A) Validation of USP30 deletion in hESCs. The indicated gRNA, which targets both isoform 1 and 2 of USP30 (exon 5 and exon 2, respectively) was used to produce a frameshift in both alleles of USP30 using Cpdf1 (Cas12) nuclease, as determined by sequence analysis of the isolated USP30 clone. Loss of USP30 protein was verified by immunoblotting of iNeuron extracts (**Figure 5B**).

(B) Heat map of the effect of USP30 deletion on the abundance of individual diGLY-containing peptides at each time point from the TMT-MS^3 data. diGLY-peptide of proteins associated with mitochondria (MitoCarta 2.0 (Calvo et al., 2016)) are with an orange box.

(C) Correlation plots of diGLY peptide TMT values for $USP30^{+/+}$ iNeurons versus $USP30^{-/-}$ iNeurons at the indicated time points post depolarization.

Figure S6

A

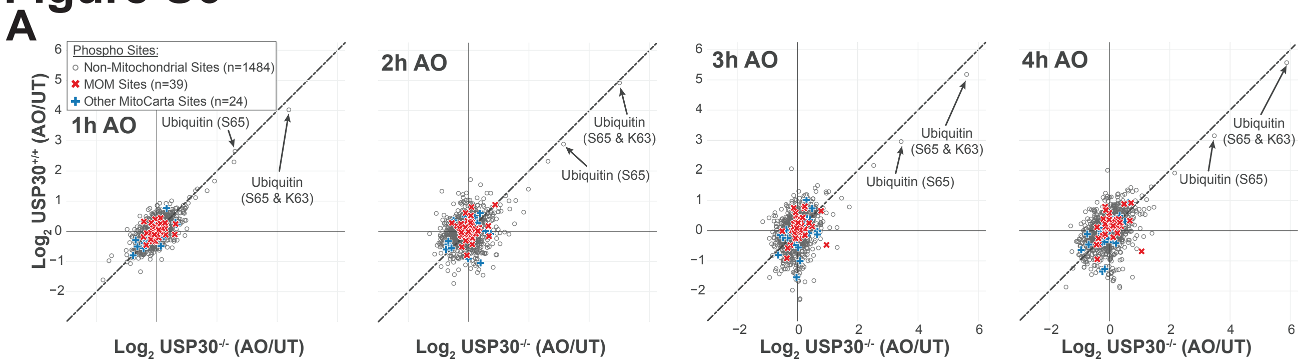

B

Quantified Phospho Peptide	
Total	2361
Unique	1547
Localized	944
Norm to Prot	1289
MitoCarta 2.0	48
MOM	39

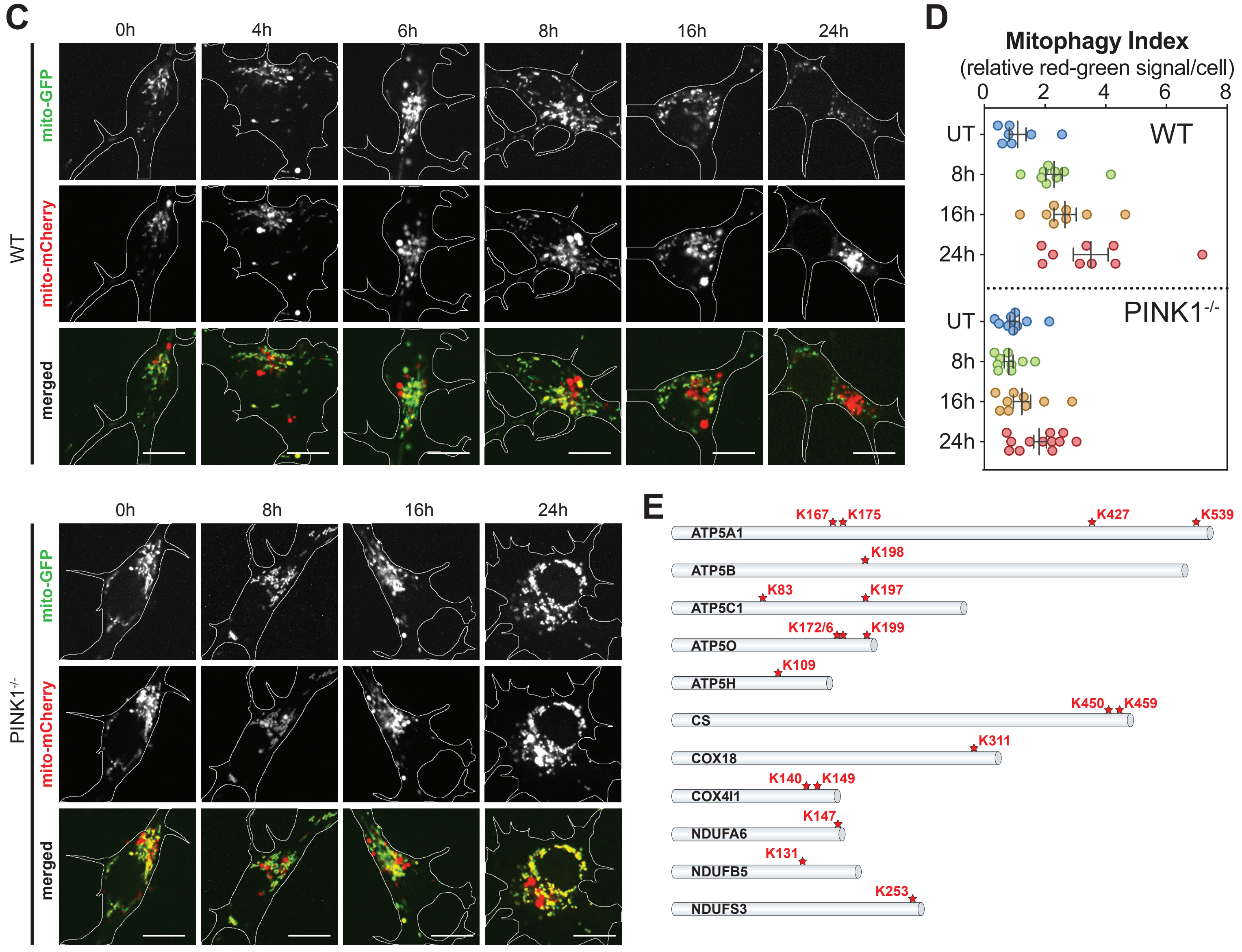

F

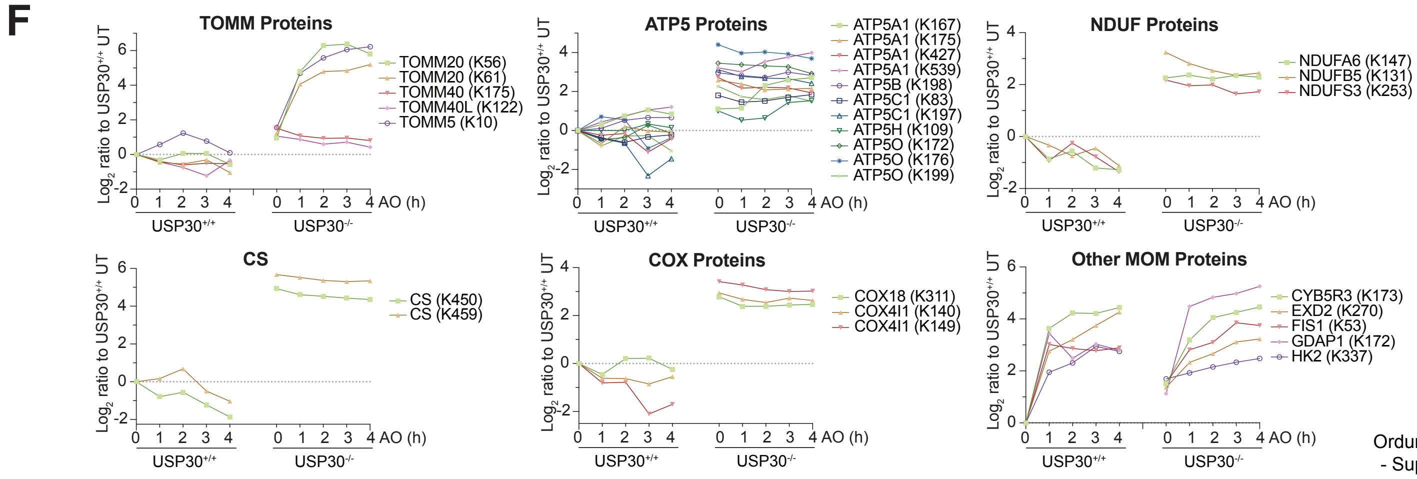

FIGURE S6. Analysis of the effect of USP30 deficiency on mitochondria in response to depolarization. Related to Figure 6.

(A) Correlation plots of phospho-peptide TMT values for USP30$^{+/+}$ iNeurons versus USP30$^{-/-}$ iNeurons at the indicated time points post depolarization.

(B) Summary of phospho-peptides quantified in the experiment described in **Figure 5A**.

(C, D) Live-cell imaging time course in iNeurons expressing mtx-QC^{XL}. Cells were depolarized with 0.5μM Antimycin A/0.5μM Oligomycin A and cells imaged (panel C) and the mitophagic flux index (panel D) as described in **STAR METHODS**.

(E) Position(s) of the ubiquitylation site, constitutively upregulated in USP30$^{-/-}$, in a cohort of substrates.

(F) Ubiquitylation kinetics for individual diGLY sites that are elevated 2-fold or more in USP30$^{-/-}$ iNeurons relative to WT cells under basal (untreated) conditions.

Figure S7

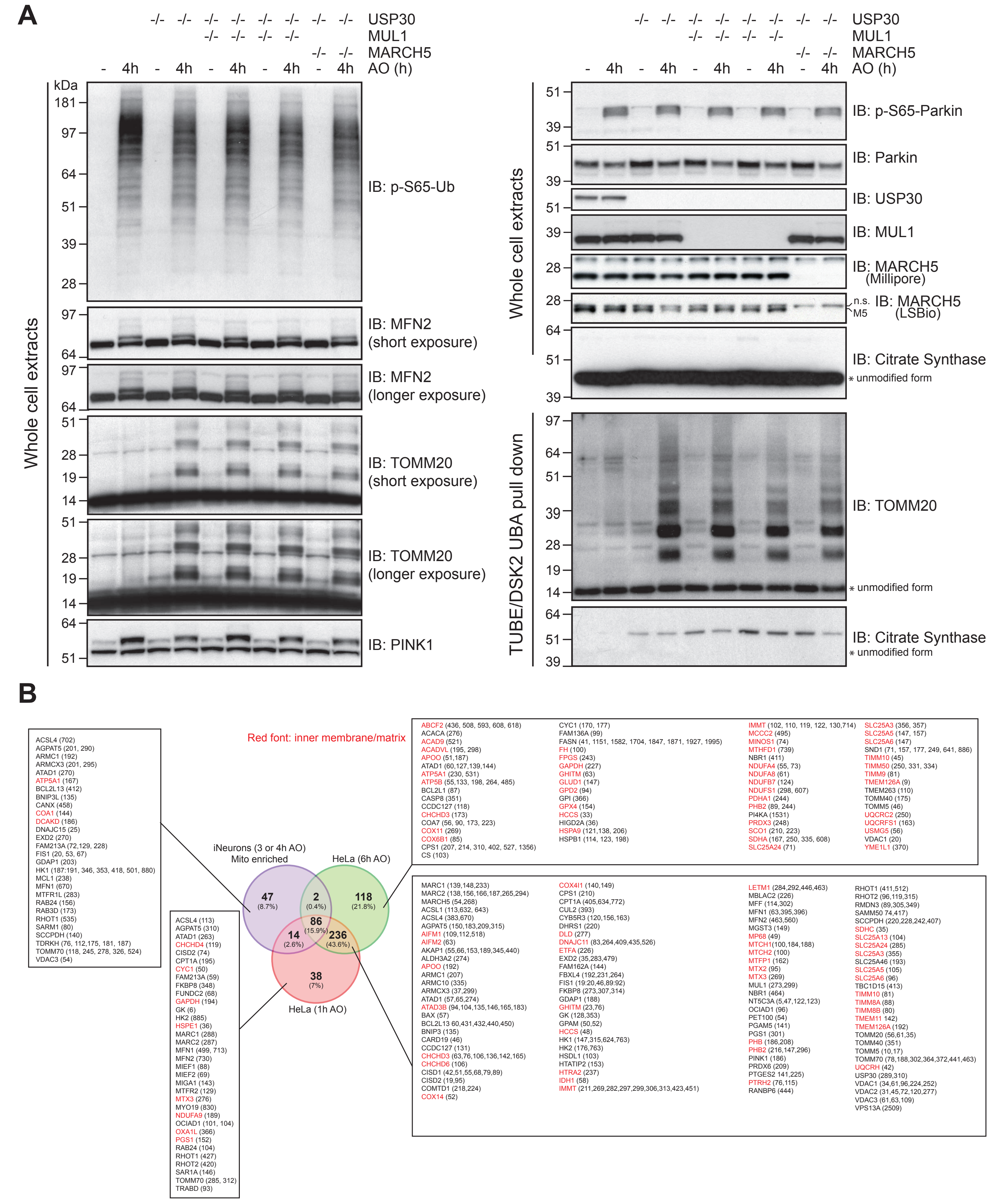
A
USP30
MUL1
MARCH5
AO (h)
kDa
IB: p-S65-Ub
IB: MFN2 (short exposure)
IB: MFN2 (longer exposure)
IB: TOMM20 (short exposure)
IB: TOMM20 (longer exposure)
IB: PINK1
Whole cell extracts
IB: p-S65-Parkin
IB: Parkin
IB: USP30
IB: MUL1
IB: MARCH5 (Millipore)
n.s.
M5
IB: MARCH5 (LSBio)
IB: Citrate Synthase
* unmodified form
IB: TOMM20
* unmodified form
IB: Citrate Synthase
* unmodified form
TUBE/DSK2 UBA pull down
B
Red font: inner membrane/matrix
iNeurons (3 or 4h AO) Mito enriched
HeLa (6h AO)
HeLa (1h AO)
47 (8.7%)
2 (0.4%)
118 (21.8%)
86 (15.9%)
14 (2.6%)
236 (43.6%)
38 (7%)
ACSL4 (702)
AGPAT5 (201, 290)
ARMC1 (192)
ARMCX3 (201, 295)
ATAD1 (270)
ATP5A1 (167)
BCL2L13 (412)
BNIP3L (135)
CANX (458)
COA1 (144)
DCAKD (186)
DNAJC15 (25)
EXD2 (270)
FAM213A (72,129, 228)
FIS1 (20, 53, 67)
GDAP1 (203)
HK1 (187:191, 346, 353, 418, 501, 880)
MCL1 (238)
MFN1 (670)
MTFR1L (283)
RAB24 (156)
RAB3D (173)
RHOT1 (535)
SARM1 (80)
SCCPDH (140)
TDRKH (76, 112,175, 181, 187)
TOMM70 (118, 245, 278, 326, 524)
VDAC3 (54)
ACSL4 (113)
AGPAT5 (310)
ATAD1 (263)
CHCHD4 (119)
CISD2 (74)
CPT1A (195)
CYC1 (50)
FAM213A (59)
FKBP8 (348)
FUNDC2 (68)
GAPDH (194)
GK (6)
HK2 (885)
HSPE1 (36)
MARC1 (288)
MARC2 (287)
MFN1 (499, 713)
MFN2 (730)
MIEF1 (88)
MIEF2 (69)
MIGA1 (143)
MTFR2 (129)
MTX3 (276)
MYO19 (830)
NDUFA9 (189)
OCIAD1 (101, 104)
OXA1L (366)
PGS1 (152)
RAB24 (104)
RHOT1 (427)
RHOT2 (420)
SAR1A (146)
TOMM70 (285, 312)
TRABD (93)
ABCF2 (436, 508, 593, 608, 618)
ACACA (276)
ACAD9 (521)
ACADVL (195, 298)
APOO (51,187)
ATAD1 (60,127,139,144)
ATP5A1 (230, 531)
ATP5B (55,133, 198, 264, 485)
BCL2L1 (87)
CASP8 (351)
CCDC127 (118)
CHCHD3 (173)
COA7 (56, 90, 173, 223)
COX11 (269)
COX6B1 (85)
CPS1 (207, 214, 310, 402, 527, 1356)
CS (103)
CYC1 (170, 177)
FAM136A (99)
FASN (41, 1151, 1582, 1704, 1847, 1871, 1927, 1995)
FH (100)
FPGS (243)
GAPDH (227)
GHITM (63)
GLUD1 (147)
GPD2 (94)
GPI (366)
GPX4 (154)
HCCS (33)
HIGD2A (36)
HSPA9 (121,138, 206)
HSPB1 (114, 123, 198)
IMMT (102, 110, 119, 122, 130,714)
MCCC2 (495)
MINOS1 (74)
MTHFD1 (739)
NBR1 (411)
NDUFA4 (55, 73)
NDUFA8 (61)
NDUFB7 (124)
NDUFS1 (298, 607)
PDHA1 (244)
PHB2 (89, 244)
PI4KA (1531)
PRDX3 (248)
SCO1 (210, 223)
SDHA (167, 250, 335, 608)
SLC25A24 (71)
SLC25A3 (356, 357)
SLC25A5 (147, 157)
SLC25A6 (147)
SND1 (71, 157, 177, 249, 641, 886)
TIMM10 (45)
TIMM50 (250, 331, 334)
TIMM9 (81)
TMEM126A (9)
TMEM263 (110)
TOMM40 (175)
TOMM5 (46)
UQCRC2 (250)
UQCRFS1 (163)
USMG5 (56)
VDAC1 (20)
YME1L1 (370)
MARC1 (139,148,233)
MARC2 (138,156,166,187,265,294)
MARCH5 (54,268)
ACSL1 (113,632, 643)
ACSL4 (383,670)
AGPAT5 (150,183,209,315)
AIFM1 (109,112,518)
AIFM2 (63)
AKAP1 (55,66,153,189,345,440)
ALDH3A2 (274)
APOO (192)
ARMC1 (207)
ARMC10 (335)
ARMCX3 (37,299)
ATAD1 (57,65,274)
ATAD3B (94,104,135,146,165,183)
BAX (57)
BCL2L13 60,431,432,440,450)
BNIP3 (135)
CARD19 (46)
CCDC127 (131)
CHCHD3 (63,76,106,136,142,165)
CHCHD6 (106)
CISD1 (42,51,55,68,79,89)
CISD2 (19,95)
COMTD1 (218,224)
COX14 (52)
COX4I1 (140,149)
CPS1 (210)
CPT1A (405,634,772)
CUL2 (393)
CYB5R3 (120,156,163)
DHRS1 (220)
DLD (277)
DNAJC11 (83,264,409,435,526)
ETFA (226)
EXD2 (35,283,479)
FAM162A (144)
FBXL4 (192,231,264)
FIS1 (19:20,46,89:92)
FKBP8 (273,307,314)
GDAP1 (188)
GHITM (23,76)
GK (128,353)
GPAM (50,52)
HCCS (48)
HK1 (147,315,624,763)
HK2 (176,763)
HSDL1 (103)
HTATIP2 (153)
HTRA2 (237)
IDH1 (58)
IMMT (211,269,282,297,299,306,313,423,451)
LETM1 (284,292,446,463)
MBLAC2 (226)
MFF (114,302)
MFN1 (63,395,396)
MFN2 (463,560)
MGST3 (149)
MP68 (49)
MTCH1(100,184,188)
MTCH2 (100)
MTFP1 (162)
MTX2 (95)
MTX3 (269)
MUL1 (273,299)
NBR1 (464)
NT5C3A (5,47,122,123)
OCIAD1 (96)
PET100 (54)
PGAM5 (141)
PGS1 (301)
PHB (186,208)
PHB2 (216,147,296)
PINK1 (186)
PRDX6 (209)
PTGES2 141,225)
PTRH2 (76,115)
RANBP6 (444)
RHOT1 (411,512)
RHOT2 (96,119,315)
RMDN3 (89,305,349)
SAMM50 74,417)
SCCPDH (220,228,242,407)
SDHC (35)
SLC25A13 (104)
SLC25A24 (285)
SLC25A3 (355)
SLC25A46 (193)
SLC25A5 (105)
SLC25A6 (96)
TBC1D15 (413)
TIMM10 (81)
TIMM8A (88)
TIMM8B (80)
TMEM11 142)
TMEM126A (192)
TOMM20 (56,61,35)
TOMM40 (351)
TOMM5 (10,17)
TOMM70 (78,188,302,364,372,441,463)
UQCRH (42)
USP30 (289,310)
VDAC1 (34,61,96,224,252)
VDAC2 (31,45,72,120,277)
VDAC3 (61,63,109)
VPS13A (2509)

FIGURE S7. Mitochondria resident E3 ligases MUL1 and MARCH5 do not modify USP30$^{-/-}$ phenotype and detailed landscape of Parkin-dependent ubiquitylation across cell lines. Related to Figure 7.

(A) The indicated iNeurons were either left untreated or depolarized (10μM Antimycin A/5μM Oligomycin A) for 4h prior to analysis of cell extracts by immunoblotting using the indicated antibodies. To analyze CS ubiquitylation, whole cell extracts were used for ubiquitin enrichment using immobilized ubiquitin binding domain (UBA) fusion proteins and bound proteins subjected to immunoblotting.
(B) Venn diagram (from **Figure 7B**) is expanded to show the identity of individual diGLY sites that were identified in each of the indicated experiments. The overlapping 86 proteins found in all 3 experiments are shown in **Figure 7C**. Sites in red indicate proteins located in either the inner mitochondrial membrane or the matrix.
